# Immunotherapy of Malignant Tumors in the Brain: How Different from Other Sites?

**DOI:** 10.3389/fonc.2016.00256

**Published:** 2016-12-07

**Authors:** Valérie Dutoit, Denis Migliorini, Pierre-Yves Dietrich, Paul R. Walker

**Affiliations:** ^1^Laboratory of Tumor Immunology, Center of Oncology, Geneva University Hospitals and University of Geneva, Geneva, Switzerland; ^2^Oncology, Center of Oncology, Geneva University Hospitals and University of Geneva, Geneva, Switzerland

**Keywords:** brain tumors, glioma, tumor immunotherapy, tumor microenvironment, brain homing

## Abstract

Immunotherapy is now advancing at remarkable pace for tumors located in various tissues, including the brain. Strategies launched decades ago, such as tumor antigen-specific therapeutic vaccines and adoptive transfer of tumor-infiltrating lymphocytes are being complemented by molecular engineering approaches allowing the development of tumor-specific TCR transgenic and chimeric antigen receptor T cells. In addition, the spectacular results obtained in the last years with immune checkpoint inhibitors are transfiguring immunotherapy, these agents being used both as single molecules, but also in combination with other immunotherapeutic modalities. Implementation of these various strategies is ongoing for more and more malignancies, including tumors located in the brain, raising the question of the immunological particularities of this site. This may necessitate cautious selection of tumor antigens, minimizing the immunosuppressive environment and promoting efficient T cell trafficking to the tumor. Once these aspects are taken into account, we might efficiently design immunotherapy for patients suffering from tumors located in the brain, with beneficial clinical outcome.

The immune system, thanks to its power and specificity, has extraordinary potential to achieve long-lasting tumor remissions, with no side effects on normal tissues. Manipulating the immune system to achieve such a goal is the objective of cancer immunotherapy, which has been under intense investigation for more than 20 years, with some successes, but also room for improvement. In particular, T cell immunotherapy aims to generate, *in vivo* or *in vitro*, efficient tumor-specific T cells able to reach the tumor microenvironment and provide long-term antitumor function. This approach comes with many complexities, namely the choice of a tumor antigen, the source of tumor-specific T cells, the need to elicit strong immune responses, and to target the immunosuppressive tumor microenvironment. Immunotherapy has been developed for many malignancies, which now includes tumors in the brain. Decades of research have helped understanding the fundamentals of immune responses to tumors and showed that tumor-specific immune responses were able to occur, but were limited by the mechanism of tumor immunoediting (1). These studies also revealed that antitumor immune responses were able to occur in the brain, following similar rules to those applying to peripheral organs (2). However, the brain, as an immune specialized site, is endowed with additional hurdles to overcome before efficient immunotherapy can be achieved. Here, the means and requirements for successful immunotherapy will be identified and potential additional requisites for efficient immunotherapy of tumors located in the brain will be discussed. Ongoing immunotherapeutic clinical trials will finally be described to appreciate the current status of these approaches.

## Tumor Immunotherapy: Current Approaches

The aim of T cell-based tumor immunotherapy is to provide patients with tumor-specific T lymphocytes that will patrol the body to detect and kill tumor cells. This can be accomplished by either active or passive approaches.

### Therapeutic Vaccination

Therapeutic vaccination relies on the patient’s immune system to react to an injected tumor vaccine. Tumor vaccines aim to raise an immune response against tumor antigens using specific peptides, proteins, tumor cells (including lysates and eluates), mRNA, or DNA, in some cases pulsed onto dendritic cells (DCs) (3). One major advantage of peptide vaccines is that the antigen is well characterized, ensuring a precise targeting of the tumor with possibly little damage to normal tissue. In this regard, the best tumor antigen is a tumor-specific antigen (TSA), resulting from a tumor-specific mutation. Whereas such TSA are the ideal targets, they are not shared by the majority of patients and were until recently not frequently exploited for peptide vaccines. However, advances in personalized vaccine approaches will most probably revive their use, as patient-specific tumor mutations can now be relatively easily identified and used as vaccine antigens (4). In contrast to TSA, tumor-associated antigens (TAAs) are shared by a larger proportion of patients and have been widely used in cancer vaccines over the years. TAA derive from proteins overexpressed in cancer cells but retaining some expression in healthy tissues, which varies depending on the antigen. This is the major drawback to their use, as potential harm to normal cells cannot be excluded, which can be fatal depending on the cells or organ involved. Although TAA-based peptide vaccines have not shown major toxicity thus far (3), adoptive cell therapy has been faced with severe adverse events including deaths due to TAA expression by normal tissues (5), as will be discussed later. Another advantage of TAA is that they are shared among patients and can thus be exploited to design multipeptide vaccines with the aim to prevent tumor escape by antigen downregulation, a phenomenon observed occasionally with single-peptide vaccination (6, 7). It is hypothesized that the latter can be circumvented by the process of epitope spreading, whereby immune responses are directed toward additional tumor antigens liberated from lysis of the initially targeted cells (8, 9). Nonetheless, the use of well-defined antigens is limited by the need for identification and many groups have therefore chosen to vaccinate with whole tumor cells or tumor mRNA (10, 11). This approach has the advantage of providing patient-specific and multiple tumor antigens for vaccination but also presents the risk of inducing immune responses to non-tumor antigens present in the preparation. In addition, the requirement for sufficient tumor for vaccine preparation restricts their use to a subset of patients in malignances where small tumor samples are received, as is the case for tumors in the brain. To overcome this hurdle, some trials are using allogenic tumor cell lines for vaccine preparation (12). Finally, the use of undefined vaccine antigens makes immunomonitoring challenging, possibly hindering correlation between vaccine-induced immune responses and clinical outcome. Regardless of the antigen source, peptide and tumor vaccines have been injected with or without DC, the latter being used to bridge innate and adaptive immunity and more efficiently initiate vaccine-specific immune responses (13).

### The Need for Adjuvants

Most antigens used in tumor vaccine are derived from self-proteins and therefore are not recognized by pattern recognition receptors of innate immunity (14). Therefore, in most ongoing clinical trials, tumor vaccines are injected with an adjuvant, which aims at stimulating innate immunity and augmenting vaccine immunogenicity. Many different adjuvants have been used since the beginning of cancer vaccine administration, but the current development of more and more ligands for innate pathogen recognition receptors such as TLR, RLR, or STING ligands, among others, is likely to improve vaccine efficacy (15). TLR and RLR are sensors that detect viral/bacterial DNA or RNA, or bacterial, fungal, or protozoan lipoproteins/peptidoglycans and induce type I interferons. Synthetic TLR3, TLR4, TLR7, and TLR9 ligands are being tested in cancer patients as single agent or in combination with cancer vaccines (15) and ligands for other TLRs are in development. STING ligands induce type I interferon after detection of intracellular DNA and have shown impressive antitumor effect in preclinical models (16–18), which should stimulate rapid translation into the clinic. In addition to the use of adjuvants, it was shown that inducing inflammation at the vaccine site by vaccination with recall antigens (tetanus and diphtheria toxoids, Td) prior to tumor antigen DC vaccine improved patient survival by increasing DC migration to the vaccine draining LN, a process which was dependant on CCL3 (19).

### T Cell Therapy

T cell therapy does not rely on patient vaccination but on the adoptive transfer of high numbers of autologous tumor-specific T cells. The latter can be generated from tumor-infiltrating lymphocytes (TIL) or from antigen-specific T cells enriched from peripheral blood. Alternatively, peripheral T cells can be engineered to express a high-avidity tumor-specific TCR (TCR-transgenic T cells) or an antibody fragment [chimeric antigen receptor (CAR) T cells] (20). Adoptive transfer with TIL is based on the demonstration that T cells found at the tumor site are tumor-specific and endowed with tumor killing activity, reflected by the fact that, in many malignancies, infiltration by activated CD8 T cells correlates with patient outcome (21). However, few tumors are highly immunogenic and thus infiltrated by lymphocytes. In addition, the fact that tumor-derived T cells might be exhausted and might not persist long enough after injection for efficient tumor eradication has prompted the development of adoptive transfer with modified peripheral blood T cells (22). One option is TCR-engineered T cells that are made to express the α and β chains of a high affinity well-characterized HLA-restricted tumor-specific TCR; these can be relatively rapidly generated and infused to any patient sharing the cognate HLA and expressing the specific tumor antigen (23). An alternative approach is CAR T cells that are engineered to express a tumor-specific antibody as a single chain fragment to redirect T cell recognition to the tumor (24). They are not HLA-restricted as their moiety for antigen recognition is an antibody and can therefore be given to any patient expressing the cognate antigen; an additional benefit is that this overcomes the mechanism of tumor evasion by MHC downregulation. One advantage of TCR-transgenic and CAR T cell transfer is that the large majority of the infused cells are tumor-specific, which provides the patient with considerable numbers of tumor-reactive cells. In addition, the antigen recognition domain of TCR-transgenic and CAR T cells can be mutated to increase affinity to the antigen, making infused cells of high avidity to the target antigen. Another key advantage of cell therapy with genetically modified T cells is the possibility to optimize the T cell product in terms of *in vivo* cell persistence, resistance to T regulatory T (Treg) cells, and effector functions (25). At the same time, increasing avidity and efficiency of infused cells renders the choice of antigen even more critical. As mentioned above, the level of adverse events observed in clinical trials using TCR-transgenic or CAR T cells is high and these can be fatal (5). On-target, off-tumor toxicities due to recognition by TAA-specific TCR-transgenic T cells of antigen expressed on healthy tissues are observed in the majority of patients treated (26). Severe adverse events due to cross-recognition of non-targeted antigens by high affinity mutated TCRs were also observed (27). To safeguard against this, many construct used to generate CARs now incorporate a suicide gene, with the aim to quickly deplete the transfused cells if life-threatening toxicity is seen. Cytokine storm, which is an early and potentially fatal adverse event resulting from the rapid activation of transferred T cells can usually be managed *via* treatment with anti-IL-6 antibodies (28). Another appealing solution was recently offered by the publication of a proof-of-concept study in mice illustrating eradication of established solid tumors by transfer of high-avidity TCR-transgenic T cells specific for one single neoepitope (29). Hence, the development of mutation-specific TCR-modified cells, even if targeting a single epitope, could allow the design of safe and powerful clinical trials by inducing epitope spreading, as seen with other tumor-specific cell therapies (30, 31).

### The Challenge of the Tumor Microenvironment

One of the greatest hurdles for efficient tumor immunotherapy is the fact that tumor-specific T cells have to exert their effector function in a hypoxic environment, in which chronic inflammation and tumor cells stimulate immunosuppression (32). Among the many mechanisms evolved by the tumor to escape immune response are the secretion of immunosuppressive cytokines (TGF-β and IL-10, among others), the recruitment or induction of immunosuppressive cells [Tregs, myeloid-derived suppressor cells (MDSCs), tumor-associated macrophages (TAMs)], the depletion of essential nutrients [by indoleamine dioxygenase (IDO) and arginase] and the expression of inhibitory molecules (FasL, PD-L1). Treg constitute an important fraction of tumor-infiltrating CD4^+^ T cells and inhibit tumor-reactive T cells either by direct cell contact or through TGF-β and IL-10 production (33). TAMs contribute to IL-10 and TGF-β production, to Treg recruitment by the secretion of CCL22, and promote tumor growth and invasion through production of endothelial growth factor, vascular endothelial growth factor (VEGF), and platelet-derived growth factor (PDGF), among others (34). MDSCs mostly act by inhibiting T and NK cell function through arginine depletion and production of nitric oxide and reactive oxygen species (35). Tumors also evade immune recognition by downregulating molecules required for T cell recognition, such as MHC, the antigen itself, or molecules implicated in antigen processing (32). Targeting these mechanisms is required to fully benefit from the efficacy of vaccine-induced or modified tumor-specific T cells.

### Immune Checkpoint Inhibitors

The immune checkpoint molecules expressed during normal immune responses to prevent immune overactivation are also playing a substantial role in antitumor immunity. Many of these molecules are expressed in tumor-specific T cells, probably due to chronic antigen stimulation occurring at the tumor site, and their expression correlates with an exhausted phenotype and loss of effector function (36). On the other hand, ligands for many immune checkpoint molecules are upregulated in the tumor environment by tumor cells, stromal cells, DCs, or MDCS and participate in antitumor response inhibition (37, 38). The physiological relevance of immune checkpoint molecules is supported by the outstanding clinical efficacy of immune checkpoint blockade (ICB) antibodies (39). Anti-CTLA4 and PD1 antibodies are now approved for several malignances and are being tested for virtually all tumor types together with anti-PD-L1 antibodies, and antibodies targeting Tim3 and LAG3 are in clinical trials, mostly in combination with anti-PD1 antibodies.

Immune checkpoint inhibitors work by allowing pre-existing immune responses to TAA or TSA to occur. However, efficacy of anti-CTLA4 and anti-PD1 as single agents has been greatest in malignancies that harbor a high rate of mutation, such as melanoma and some lung carcinoma (40, 41), suggesting that TSA-directed immune responses are prevalent. Accordingly, studies in melanoma have shown that the majority of tumor-reactive T cells found in TILs were recognizing TSA and not TAA (42) and response to ICB has been shown to be associated with detection of neoepitope-specific T cell responses (40). A critical question for the use of ICB for malignancies harboring low rates of mutations is thus to be able to determine the minimal mutation load required to achieve efficient tumor destruction with these agents. In that regard, one study in melanoma showed that patients harboring tumors with >100 mutations were more prone to benefit from anti-CTLA4 treatment (40), suggesting a threshold for ICB molecule efficacy. Nonetheless, this is not absolute, as some patients still benefit from treatment with IBC despite low-mutation rate (43). In addition to allowing neoantigen-specific immune responses to occur, immune checkpoint inhibitors are also able to amplify vaccine-induced immune responses and trials of peptide vaccination against melanoma antigens in combination with a soluble LAG3 have been reported, which showed the safety of the approach (44, 45). Combination with other immunotherapies is ongoing and is likely to be an important contribution of ICB antibodies to cancer treatment in the future.

## Immune Responses to Tumors Arising in the Brain

With the exception of primary central nervous system (CNS) lymphoma (PCNSL) arising from B cell transformation, most primary brain tumors (astrocytoma, oligodendroglioma, oligoastrocytoma, and ependymoma) derive from glial cells. They account for approximately 2% of all cancers, but the associated mortality is very high, the 5-year survival rates for grade III astrocytoma and glioblastoma (GBM, grade IV), the most common, being 30 and 3%, respectively (46). The major characteristics of GBM are its highly invasive nature and extraordinarily low rate of metastasis outside the brain. Regarding PCNSL, it is a rare disease, representing about 2% of all primary brain tumors in immunocompetant hosts (47), but, similar to gliomas, is very aggressive and associated with poor prognosis. Likewise, it also exceptionally metastasizes outside the brain, for reasons that are not yet clear.

Although it was long thought that the brain was an immune sanctuary, it is now established that immune responses toward tumors located in the CNS are able to occur. This is substantiated by both animal models of intracranial tumors, which show that strong antitumor immune responses are able to control tumor cells (48, 49), and by observations in humans revealing that T cells are detected at the tumor site and positively influence survival (50–52). Antigen-specific spontaneous B and T cell immune responses have been detected in patients with glioma (53–56), although less frequently than in other malignancies such as melanoma. PCNSL are associated with a robust inflammatory response, including infiltrating activated macrophages and reactive T cells, the latter being associated with a favorable outcome (57, 58).

The mechanisms of immune system activation by tumors located in the brain have been explored in the last decades. Features of the brain, which are different from other sites, namely lack of conventional lymphatic draining, absence of resident DCs in the brain parenchyma, and existence of the blood–brain barrier, are no longer regarded as obstacles to initiation of immune responses but might present a high threshold to be reached before efficient spontaneous antitumor immunity is induced. In spite of these, it has been shown that antitumor immune responses were able to occur. Antigens are able to drain from the brain parenchyma to reach the cervical lymph nodes (59, 60) where they are presented by DCs to T cells (61), leading to the proliferation of tumor-specific cells that will be able to home to the brain *via* expression of, among other molecules still to be discovered, VLA4/α4β1 and CXCR3 (62, 63). These T cells are retained at the tumor site *via* expression of αEβ7 (62) and could potentially represent tissue-resident memory cells poised to be reactivated upon re-encounter of tumor-expressed antigens (64).

However, brain tumors, similar to tumors arising in other sites, are able to resist immune attack through various means including MHC downregulation (65), release of immunosuppressive cytokines such as TGF-β (66), VEGF (67), prostaglandin E2 (68), IL-10 (69), and of enzymes such as IDO (70) and arginase (71), attraction of Tregs (72), and MDSCs (71, 73). In particular, Tregs have been shown in mice models of spontaneous glioma to be present at the tumor site very early, even before symptoms are visible (35). IDO, which can inhibit conventional T cells and induce Tregs, is expressed virtually in all GBM and level of expression is associated with poor prognosis (70). In addition, GBM can induce apoptosis of activated T cells through expression of FasL (74) and PD-L1, the latter being expressed by GBM cells but also by TAMs (75, 76) and able to inhibit glioma-infiltrating lymphocytes, which commonly express PD-1 (77). Finally, hypoxia is associated with poor clinical outcome in GBM patients (78). All these parameters converge to attenuate spontaneous immune responses occurring in patients with brain tumors, leading to inefficient tumor control.

In addition to that, intrinsic differences between the brain and peripheral organs exist, which might lead to suboptimal immune activation against tumors located in the brain as compared to tumors located in peripheral organs (79). These differences certainly need to be considered when designing immunotherapeutic strategies for tumors in the brain.

### Priming of Immune Responses to Brain Tumor Antigens

As described above, initiation of immune response to antigens located in the brain occurs, antigen presentation to naïve T cells occurring either *via* drainage of soluble antigen to LN or by transport *via* emigration of antigen-bearing DCs (62, 80). Immune response elicited by antigens that drain predominantly to the cervical LN were shown to be less effective than responses elicited to the same antigen reaching other lymph nodes (81), potentially due to induction of immunosuppressive myeloid cells. This might lead to suboptimal induction of immune response to tumors located in the brain as compared to other sites. Nonetheless, it cannot be entirely excluded that immune responses to brain tumors are elicited in the periphery in response to circulating tumor cells reaching secondary lymphoid organs; these having been reported in a significant number of patients with GBM (82, 83).

These issues need to be taken into consideration for therapeutic vaccination. A very important concern for tumor vaccines is the site of antigen injection to prime antitumor immune responses. In the many clinical trials of peptide and tumor vaccination performed in the last decades, several injection sites have been used, precluding evaluation of the efficacy of vaccination from different sites. However, a preclinical study comparing injection of a model antigen at different sites in glioma-bearing mice was able to demonstrate that vaccinating far away from the tumor was best to induce optimal CD8 effector function and brain infiltration (84). This was due to tumor-derived immunosuppressive factors reaching the LN and influencing the T cell response. These results are compatible with spontaneous antitumor immunity discussed previously (81).

### Homing to the Brain

Tumor-specific T cells generated by vaccination or adoptive cell transfer need to reach the brain in order to exert their effector function. During a spontaneous antitumor immune response, homing of T cells to the tumor site is determined at the site of antigen capture by the APC, which will imprint T cells during priming in the lymph nodes (62). Regarding the CNS, it was shown that T cell expression of α4β1 and CXCR3 facilitated infiltration of the brain (62, 63). It is therefore important to replicate this brain homing phenotype during therapeutic vaccination and adoptive cell transfer in order for sufficient cells to reach the brain. Indeed, it has been shown in animal models that adoptively transferred T cells are less efficient at infiltrating the brain than peripheral sites (85). Similarly, although adoptive transfer of TIL was shown to mediate regression of melanoma brain metastases (86), the latter have been shown to be less infiltrated by CD3^+^ T cells than extracerebral metastatic sites, suggesting lower brain T cell homing (87). Therefore, for vaccine-induced or adoptively transferred cells to reach the brain, additional interventions need to be made. Brain homing has been shown to be enhanced by CXCL10, one of the CXCR3 ligands, secreted at the tumor environment (88), which can be promoted by injection of poly-ICLC (polyriboinosinic–polyribocytidylic acid stabilized with poly-l-lysine and carboxymethylcellulose), a TLR3 agonist. TLR3 is the most abundant TLR expressed by astrocytes and microglial cells and its activation has been shown to induce pro-inflammatory cytokines such as TNF-α, IL-6 and IFN-β and chemokines such as CCL2, CCL5, and CXCL10 (89). As a consequence, poly-ICLC has been extensively tested in patients with glioma, with the reported induction of robust vaccine-specific CD8 T cell responses associated with detection of CXCL10 in the circulation (90, 91). Regarding adoptive cell transfer, choosing culture conditions to generate cells with a tumor homing phenotype may be possible, although the exact conditions for this are not yet defined. In addition, transgenic expression of selected chemokine receptors could be envisaged in the case of TCR-transgenic and CAR T cells (92), although these strategies remain in the preclinical phase at present. Alternatively, it has been shown that increased brain migration of adoptively transferred CD8 T cells can be obtained by co-infusion of CD4 T cells specific for the same tumor antigen and bearing the Th1 phenotype (93).

### Effector Function in the Brain Immunosuppressive Environment

Even if we know that immune response are able to occur in the brain, this organ nonetheless tightly regulates inflammation, mostly through TGF-β secretion. TGF-β2 is the most abundant TGF-β isoform detected in the adult brain and modulates response to brain lesions, including blocking of several pro-inflammatory cytokines and of MHC class II upregulation (94). In addition, the brain is one of the most densely vascularized organs in the body with VEGF being the main inducer of angiogenesis. As stated before, VEGF is also a strong inducer of immunosuppression by mediating accumulation of MDSC and Tregs and inhibiting the function and migration of T lymphocytes to the tumor (95). In consequence, tumor-specific T cells elicited by immunotherapy have to overcome, once they reach the brain, a series of obstacles before they can exert their effector function. As indicated before, CNS cells express FasL, which will induce apoptosis of incoming Fas^+^ T cells (96). Surviving cells will have to cope with the immunosuppressive factors and cells described above and will further be inhibited by PD-L1 expression by tumor and myeloid cells. All these factors need to be considered to design efficient immunotherapies.

### Efficacy of Immunotherapies

Recently, the distinction of T cell inflamed versus non-T cell inflamed tumors has allowed stratifying patients according to prognosis and response to immune checkpoint inhibitors (97). The current understanding is that, in T cell inflamed tumors, recruitment of tumor-specific CD8 T cells leads to secretion of pro-inflammatory (mostly IFN-γ) cytokines, which stimulates upregulation of PD-L1 and IDO and recruitment of Tregs (98, 99). In non-T cell inflamed tumors, T cell markers and chemokines involved in T cell recruitment are not detected, possibly due to lack of priming of the antitumor response or/and lack of migration at the tumor site. Importantly, T cell inflamed tumors have been shown to be associated with response to both therapeutic vaccines (100) and checkpoint blockade (98, 101, 102). In this regard, GBM can be considered as a poorly T cell inflamed tumor, as compared to tumors located in peripheral organs such as melanoma, renal cell carcinoma, breast, or ovarian cancers (103). Similarly, PCNSL are poorly infiltrated by immune cells as compared to their peripheral counterpart (104), suggesting that tumors located in the brain might be less prone to respond to immunotherapies, including ICB. Immunotherapeutic interventions should therefore include strategies to promote inflammation at the tumor site in the brain, possibly by inducing innate signaling to trigger antitumor adaptive immunity. One strategy to achieve this is tumor delivery of stimulator of interferon genes (STING) agonists, which have been shown in mouse models of glioma to promote infiltration by CD4 and CD8 T cells and prolong survival (18). Alternatively, type I IFN production can be induced by radiotherapy (105), and radiation of the tumor site has been shown to induce double strand DNA breaks and subsequent type I IFN activation *via* STING in mouse models of glioma (106). Finally, one study in mouse models, not yet explored for GBM, showed that treatment of non T cell inflamed tumors with LIGHT, a member of the tumor necrosis factor superfamily, led to secretion of pro-inflammatory chemokines and recruitment of T cells at the tumor site, which was associated with greater response to ICB (107).

### Choice of Antigens

The choice of antigen for designing immunotherapeutic strategies is arguably even more important for tumors located in the brain as compared to those occurring in other sites. Indeed, whereas attack of healthy cells expressing the tumor antigen to some level, such as skin depigmentation observed due to the targeting of melanoma antigens shared by melanoma cells and melanocytes, can be tolerated in some organs, this is more critical for the brain. TAA recognized by T cells have been identified in glioma, although their number is fewer than for other malignances such as melanoma. They include, among others, IL13Rα2, EphA2, WT1, and survivin (108) and the antigens composing the IMA950 peptide cocktail (56), which were eluted from the surface of GBM cells and were shown to be expressed by the majority of patients with GBM (56). Equally, few TSA have been detected to date for GBM, but more will probably be identified in the future thanks to increased use of tumor sequencing and patient-specific epitope identification (109). Until now, the most used TSA for GBM immunotherapy are EGFRvIII, a mutant antigen derived from the EGFR protein, which is expressed by 20–50% of GBM patients (110) and IDH1R132H, derived from the IDH1 protein and mainly expressed in grade II and III astrocytoma and patients with secondary GBM (111).

Altogether, tumors located in the brain have particular immunological features that will need to be taken into account for the design of immunotherapies. Among this, (i) the careful choice of antigen, (ii) the need to stimulate inflammation of the tumor site, (iii) to target the brain immunosuppressive milieu, (iv) to vaccinate far from the tumor site, and (v) to help cells home to the brain might be mandatory to address for brain tumor immunotherapy to be efficient.

## Ongoing Clinical Trials for Tumors in the Brain

Most immunotherapeutic approaches developed to date for tumors located in the brain have mostly targeted patients with glioma. PCNSL has not yet attracted much attention for vaccines and cell therapy and only one trial is investigating ICB in this malignancy. Most of the trials described below will therefore relate to glioma.

### Peptide and Tumor Vaccines

Peptide vaccines (with or without DCs) for GBM have mostly used multipeptidic TAA vaccine formulations in adjuvant, incorporating the EphA2, IL-13Rα2, WT1, and survivin (90, 91, 112), or the IMA950 cocktail (113), although some peptides have been used alone (114–116). These trials have shown that vaccine-specific immune responses were elicited, which were not associated with autoimmunity, and clinical benefit was possibly observed for some individual patients. Following these results, additional studies are being conducted (Table 1), with single peptides (NCT02455557, NCT02049489), cocktails of minimal T cell epitopes (NCT01130077, NCT02358187, NCT02078648, NCT01920191, NCT02149225, NCT02709616), mixtures of overlapping peptides (NCT02332889), or DC-pulsed mRNA (NCT02649582, NCT02529072, NCT02465268, NCT02366728). One study is addressing efficacy of vaccination in pediatric patients with ependymoma (NCT01795313). Interestingly, some trials of personalized vaccination are ongoing (NCT01814813, NCT02709616, NCT02149225), one of which selects the peptides according to peptide elution from the patient’s tumor, thus ensuring presence of the target at the tumor surface (NCT02149225).

**Table 1:** **Currently ongoing peptide and tumor vaccine trials in tumors located in the brain**.

Immunogen	Adjuvant	Additional drugs	Patient population	Diagnostic	Phase	Estimated enrollment	Country	NCT number
**Peptide vaccines**								

**Peptide alone**								

** Tumor-associated antigens (TAAs)**								

*** Single peptide***								

Long peptide from survivin-KLH	GM-CSF + montanide			Newly diagnosed glioblastoma (GBM)	II	50	USA	NCT02455557

*** Multiple peptides***								

HLA-A2-restricted peptides from EphA2, IL-13Rα2, and survivin	Poly-ICLC		Pediatric	HGG, DIPG, and recurrent LGG	Pilot	60	USA	NCT01130077

HLA-A2-restricted peptides from EphA2, IL-13Rα2, and survivin	Poly-ICLC		Pediatric	LGG	II	25	USA	NCT02358187

HLA-A2-restricted peptides from EphA2, IL-13Rα2, and survivin	Imiquimod		Pediatric	Recurrent ependymoma	na	24	USA	NCT01795313

SL-701 (HLA-A2-restricted peptides from EphA2, IL-13Rα2, and survivin)	Poly-ICLC	Bevacizumab	Adult	Recurrent GBM	I/II	76	USA	NCT02078648

IMA950 (10 HLA-A2-restricted peptides from BCAN, CSPG4, FABP7, IGF2BP3, MET, NLGN4X, NRCAM, PTPRZ1, TNC plus 2 MHC class II peptides from survivin and MET)	Poly-ICLC		Adult		I/II	16	Switzerland	NCT01920191

Personalized overexpressed HLA-A2 or -A24-restricted peptides plus mutated peptides	GM-CSF + poly-ICLC		Adult	Newly diagnosed GBM	I	20	6 centers in Europe (GAPVAC)	NCT02149225

HSPPC-96	None	Bevacizumab	Adult	Recurrent GBM	II	165	USA	NCT01814813

** Tumor-specific antigens (TSAs)**								

*** Single peptide***								

EGFRvIII peptide	GM-CSF	Bevacizumab	Adult	EGFRvIII+ recurrent GBM	II	168	USA (ReACT)	NCT01498328

EGFRvIII peptide^a^	GM-CSF		Adult	EGFRvIII+ recurrent GBM	III	700	Worldwide (ACT IV)	NCT01480479

IDH1R132H peptide	Montanide + imiquimod		Adult	IDH1R132H-mutated newly diagnosed HGG	I	39	Germany (NOA-16)	NCT02454634

IDH1R132H peptide	Montanide + GM-CSF + Td vaccine		Adult	IDH1R132H-mutated recurrent LGG	I	24	USA (RESIST)	NCT02193347

Mutated peptides	Poly-ICLC		Adult	Newly diagnosed GBM (UnMe MGMT)	I	20	USA	NCT02287428

Mutated long peptide	Poly-ICLC		Adult	Newly diagnosed GBM	Pilot	10	USA	NCT02510950

Personalized overexpressed HLA-A2 or -A24-restricted peptides plus mutated peptides	GM-CSF + poly-ICLC		Adult	Newly diagnosed GBM	I	20	6 centers in Europe (GAPVAC)	NCT02149225

**DC + peptides/mRNA**								

** TAAs**								

*** Single peptide***

ICT-121 (CD133 peptides)	None		Adult	Recurrent GBM	I	20	USA	NCT02049489

*** Multiple peptides***

Overlapping peptides from MAGE-A1, MAGE-A3, and NY-ESO-1	Poly-ICLC	Decitabine	Pediatric	HGG, PNET, and medulloblastoma	I/II	10	USA	NCT02332889

Personalized among preselected antigens	Imiquimod or Td vaccine		Adult	Newly diagnosed GBM	I/II	20	China (PERCELLVAC)	NCT02709616

*** mRNA***

WT1 mRNA	None		Adult	Newly diagnosed GBM	I/II	20	Belgium (ADDIT-GLIO)	NCT02649582

pp65 mRNA	None	Nivolumab	Adult	Recurrent HGG	I	66	USA (AVERT)	NCT02529072

pp65 mRNA	GM-CSF + Td vaccine		Adult	Newly diagnosed GBM	II	150	USA (ATTAC-II)	NCT02465268

pp65 mRNA	Td vaccine	Basiliximab	Adult	Newly diagnosed GBM	II	116	USA (ELEVATE)	NCT02366728

**Tumor vaccines**								

**Tumor alone**								

Tumor lysate from GBM6 cell line	Imiquimod		Adult	LGG	Pilot	27	USA	NCT01678352

Tumor lysate from GBM6 cell line	Poly-ICLC		Adult	Recurrent LGG	Pilot	30	USA	NCT02549833

Tumor lysate from GBM6 cell line	Imiquimod		Pediatric	DIPG	Pilot	8	USA	NCT01400672

**DC + tumor**								

Tumor lysate	Imiquimod		Adult + pediatric	Recurrent LGG or HGG	I	20	USA	NCT01808820

Tumor lysate	Imiquimod		Pediatric	Recurrent HGG	I	20	USA	NCT01902771

Tumor lysate	Resiquimod + poly-ICLC		Adult	Newly diagnosed or recurrent HGG	II	60	USA	NCT01204684

Tumor lysate	None		Adult	Newly diagnosed or recurrent LGG	II	18	USA	NCT01635283

Tumor lysate from allogenic stem-like cell line	None	Bevacizumab	Adult	Newly diagnosed or recurrent GBM	I	40	USA	NCT02010606

Tumor lysate from autologous stem-like cells	None		Adult	Newly diagnosed GBM	II	100	China	NCT01567202

*DIPG, diffuse intrinsic pontine glioma; HGG, high-grade (III or IV) glioma; LGG, low-grade (grade II) glioma; poly-ICLC, polyinosinic–polycytidylic acid stabilized with polylysine and carboxymethylcellulose; Td, tetanus diphtheria; UnMe MGMT, unmethylated MGMT promoter*.

*^a^This study was recently discontinued after interim analysis due to absence of benefit as compared to control arm*.

Trials with TSA in glioma have mostly focused on the EGFRvIII mutation as a single peptide vaccine and two clinical trials in newly diagnosed (NCT01480479) or recurrent (NCT01498328) GBM are ongoing. However, whereas phase II studies had shown benefit for patients with recurrent or newly diagnosed GBM (7, 117, 118), the unique phase III trial assessing the benefit of EGFRvIII peptide vaccine in addition to standard treatment in newly diagnosed GBM patients (NCT01480479) was recently discontinued due to absence of improved overall survival in patients receiving the vaccine versus standard treatment.^1^ Maybe this vaccine would profit from combination with immune checkpoint inhibitors to enhance vaccine efficacy or with other peptides to prevent immune escape (7). In addition to EGFRvIII, clinical trials targeting a long peptide spanning the IDH1R132H mutation occurring in grade II/III and secondary GBM are ongoing (NCT02454634, NCT02193347). Identification of the latter epitope, which is recognized by CD4 T cells, provides the opportunity to target both CD4 and CD8 T cells by generating a composite vaccine including the IMA950 antigens and the peptide spanning the IDH1R132H mutation. Finally, three trials are assessing efficacy of vaccination with neoantigens in GBM (NCT02287428, NCT02510950, NCT02149225), one trial importantly addressing the presence of the mutated peptide at the tumor cell surface (NCT02149225).

Although some studies inject peptide or DC/peptide vaccines alone, the majority of studies inject the peptides with an adjuvant, mostly the TLR3 ligand poly-ICLC, the TLR7 ligands imiquimod and resiquimod, GM-CSF, or Montanide. Given the critical importance of adjuvant choice for therapeutic cancer vaccination revealed in preclinical studies (119–121), this issue will eventually have to be addressed in a clinical context. Interestingly, subsequent to clinical and mice studies showing that preconditioning the tumor vaccine injection site by a recall response to tetanus/diphtheria improved lymph node homing of tumor antigen-bearing DCs and magnitude of immune responses (19), four studies (NCT02193347, NCT02709616, NCT02465268, NCT02366728) use a Td recall vaccine as adjuvant, some testing as part of their clinical trial efficiency of DC migration to lymph nodes (19). Finally, one study is adding the anti-PD1 antibody nivolumab to a pp65CMV vaccine in recurrent grade III or IV glioma patients.

Vaccines using autologous tumor or allogenic GBM cell lines as source of tumor antigens are mostly employing pulsed DCs, although three pilot studies are injecting lysate from the allogenic GBM6 stem-like cell line (122) without DCs, in low-grade glioma (LGG, grade II, NCT01678352, NCT02549833) or diffuse intrinsic pontine glioma (DIPG, NCT01400672; Table 1). Trials using tumor lysate-pulsed DCs are using either autologous tumor (NCT01808820, NCT01204684, NCT01902771, NCT01635283), or stem-like cells (NCT01567202), or an allogenic stem-like cell line (NCT02010606). As for peptide vaccines, tumor cell vaccines are usually injected with one of the three above-mentioned TLR ligands, with one study combining poly-ICLC and resiquimod. As stated before, there is no trial of peptide or tumor vaccine ongoing for PCNSL.

### Cell Therapy

At least one study of TIL infusion has been performed to date in brain tumor patients (123). With regard to peripheral blood-derived antigen-specific T cell transfer, only one study is being conducted, assessing the safety and efficacy of autologous CMV pp65-specific T cells to target GBM cells potentially expressing CMV (NCT02661282) (124). This might be due to the difficulty in detecting high levels of non-viral glioma-specific T cells in the peripheral blood of glioma patients and to the difficulty of amplifying them to great numbers for reinfusion. The latter phenomenon is probably related to the systemic defects in T cell function and proliferation observed in glioma patients, which are more pronounced than in other malignancies (125). Studies using TCR-transgenic T cells incorporating TCRs from glioma-specific T cells are similarly not yet being tested in the clinical setting, most probably due to the paucity of antigen-specific T cell clones characterized thus far for glioma. One study reporting generation of antigen-specific T cell clones from patients with GBM specific for different TAA (56) might be the first step toward development of this approach as it provides T cells with exploitable TCR sequences.

Studies with CARs have, in contrast, been quite extensively tested in preclinical glioma models and are in clinical trials (126). In the last 20 years of CAR development, initial experiments using first generation CARs bearing only the CD3ζ chain as signaling domain showed that such constructs were limited in efficacy. This led to the design of constructs incorporating CD28 or 4-1BB as costimulatory molecules (second generation CARs), which resulted in impressive success for the treatment of hematological malignances (127). Third generation CARs incorporating two costimulation molecules are being tested in B cell malignancies and neuroblastoma and a few clinical trials are even testing 4th generation CARS with additional CD27 costimulation. In brain tumors, CAR studies targeting six different antigens (EGFR, EGFRvIII, EphA2, Her2, IL13Rα, and MUC1) are ongoing (NCT02331693, NCT01454596, NCT02575261, NCT02442297, NCT02208362, NCT02617134), using second (CD28 or 41BB costimulation) or third (CD28 and 41BB costimulation) generation constructs (Table 2). Of note, the IL13Rα CAR, unlike the majority of CARs that use a single chain fragment variable part (scFv) as the antigen-binding moiety, is composed of a modified IL-13 molecule (128). The safety profile of targeting some of the above-mentioned antigens is under scrutiny because of reported toxicity due to Her2 expression in heart and lung (129) and by expression of non-mutated EGFR in epithelial cells (130). Interestingly, two studies are injecting the CAR T cells in the brain, either intratumorally, in the resection cavity, or intraventricularly (NCT02442297, NCT02208362). Two other trials are using immunostimulatory cytokines, namely IL-2 with the 3rd generation EGFRvIII-specific CAR (NCT01454596) and IL-12 with the 3rd generation MUC1-specific CAR (NCT02617134, in the CAR construct itself), with the aim to enhance CAR T cell efficacy, although caution is warranted for IL-12 use (131). Finally, in an attempt to transfer CAR T cells that can best repopulate the T cell niche and generate long-term effector cells, a study targeting the IL13Rα protein is injecting central memory-enriched CAR T cells (132). Again, no cell therapy protocols are ongoing for PCNSL.

**Table 2:** **Currently ongoing cell therapy trials in tumors located in the brain**.

Specificity	Adjuvant	Additional drugs	Patient population	Diagnostic	Phase	Estimated enrollment	Country	NCT number
**Naturally occurring T cells**								

CMV-specific T cells			Adult	Newly diagnosed or recurrent HGG	I/II	54	USA	NCT02661282

**CARs**								

EGFR (CD28 costimulatory domain)		Cyclophosphamide fludarabine	Adult	Recurrent glioblastoma (GBM) with EGFR amplification	I	10	China	NCT02331693

EGFRvIII (CD28 and 41BB costimulatory domains)	IL-2	Cyclophosphamide fludarabine	Adult	EGFRvIII^+^ recurrent GBM	I/II	107	USA	NCT01454596

EphA2 (CD28 costimulatory domain)			Adult	Newly diagnosed or recurrent HGG	na	60	China	NCT02575261

Her2 (CD28 costimulatory domain)			Adult	Her2^+^ recurrent GBM	I	14	USA (iCAR)	NCT02442297

IL13Rα2 (41BB costimulatory domain)			Adult	Recurrent HGG	I	36	USA	NCT02208362

MUC1 (CD28 and 41BB costimulatory domains)	IL-12 in CAR construct	Cyclophosphamide fludarabine	Adult	MUC1^+^ recurrent GBM	I/II	20	China	NCT02617134

*HGG, high-grade (III or IV) glioma; na, not available*.

## Targeting the Tumor Environment

As discussed above, TGF-β is one of the main immunosuppressive molecules requiring targeting for tumors located in the brain. Accordingly, many trials using mRNA antisense oligonucleotides, soluble receptors, or antibodies to TGF-β and molecules inhibiting the kinase activity have been tested (133). Although reports from preclinical models were promising (66), clinical studies thus far have failed to demonstrate survival benefit associated with the use of TGF-β-targeting agents. The TGF-β mRNA antisense oligonucleotides trabedersen (AP12009) has not shown benefit in patients with grade III or IV glioma and is not being further tested (134). Galunisertib (LY2157299), a TGF-β receptor I kinase inhibitor, failed to demonstrate improved overall survival as compared to lomustine in patients with recurrent GBM (135) but is now being tested in combination with nivolumab in patients with GBM and recurrent pancreatic cancer and hepatocellular carcinoma (NCT02423343; Table 3). Similarly, fresolimumab, a pan-TGF-β antibody failed to show survival benefit in patients with glioma (136). Although these results are quite discouraging, it is important to pursue investigation of TGF-β targeting. One reason for the inefficiency of TGF-β blockade might be the activation of alternative pathways. We might therefore need to simultaneously target TGF-β and alternative pathways such as EGFR, PI3K/Akt, NF-κB, or JAK/signal transducer and activator of transcription (STAT), a strategy which has shown efficacy in preclinical studies of pancreatic tumors (137).

**Table 3:** **Currently ongoing trials targeting the tumor microenvironment**.

Target	Molecule	Additional intervention	Patient population	Diagnostic	Phase	Estimated enrollment	Country	NCT number
**TGF-β**								

	Galunisertib (TGF-β receptor I kinase Inhibitor)	Nivolumab		Glioblastoma (GBM), recurrent NSCLC, and HCC	I/II	100	USA and Spain	NCT02423343

**IDO**								

	Indoximod (D-1MT)	Bevacizumab	Adult	Recurrent HGG	I/II	144	USA	NCT02052648

	Indoximod (D-1MT)		Pediatric	Newly diagnosed HGG, ependymoma, and medulloblastoma	I	66	USA	NCT02502708

	Epacadostat	Nivolumab	Adult	Advanced solid tumors including recurrent GBM	I/II	291	USA	NCT02327078

**STAT3**								

	WP1066		Adult	Recurrent HGG, melanoma brain metastases	I/II	33	USA	NCT01904123

**MDSC**								

	Capecitabine (prodrug of 5-flourouracil)	Bevacizumab	Adult	Recurrent GBM	I	12	USA	NCT02669173

	CSF1-R inhibitor (PLX3397)			Newly diagnosed GBM	I/II	65	USA	NCT01790503

	Anti-CSF1-R antibody (FPA008)	Nivolumab	Adult	Solid tumors including GBM	I	280	USA	NCT02526017

**Tregs**								

	Basiliximab (anti-CD25)	pp65 mRNA Td vaccine	Adult	Newly diagnosed GBM	II	116	USA (ELEVATE)	NCT02366728

*d-1MT, 1-methyl-d-tryptophan; HGG, high-grade (III and IV) astrocytoma; nivolumab, fully human IgG4 anti-PD1; NSCLC, non-small cell lung cancer*.

Vascular endothelial growth factor, due to its critical role in brain tumor angiogenesis, is being targeted using different approaches. The monoclonal antibody bevacizumab is approved as a single agent for the treatment of recurrent glioma (138, 139), but did not demonstrate survival benefit for patients with newly diagnosed glioma (140, 141). It is being used in trials of therapeutic vaccination in the setting of recurrent glioma (NCT02078648, NCT01814813, NCT01498328), but is not tested *per se* in combination with other interventions. Aflibercept (VEGF Trap), a recombinant fusion protein, which acts as scavenger molecule for VEGF, improved survival in preclinical models, possibly due to its high affinity for VEGF, but failed to demonstrate antitumor activity in patients (142). A number of small molecule inhibitors of the kinase activity of VEGF receptor are being tested in glioma (including cediranib, sunitinib, pazopanib, vandetanib, and sorafenib), but not in combination with immunotherapy for the time being.

A third pathway of investigation in brain tumors is the IDO pathway, IDO being detected in virtually all glioma samples, although not normally expressed in the brain (70, 143). Studies in mouse models of glioma using the IDO inhibitor 1 methyltryptophan (1MT) suggested that combination with other molecules might be required for antitumor activity to be seen (144); however, indoximod (D-1MT) is being tested as single agent in patients with newly diagnosed (NCT02502708, pediatric population) and recurrent glioma (NCT02052648). A more recent IDO inhibitor, epacadostat (INCB24360), selectively inhibits the enzymatic activity of IDO1 and is being tested in patients with advanced solid malignancies including recurrent GBM, in combination with the anti-PD1 antibody nivolumab (NCT02327078).

Another currently targeted protein in brain tumors is STAT3, a molecule that is downstream of several oncogenic signaling cascades in glioma, including EGFR and PDGF receptor. Constitutive STAT3 activation is detected in 50–60% of high-grade glioma (145) and mediates immune suppression at the tumor site (146). It is also been shown to be activated in PCNSL (147). A trial with WP1006, an inhibitor of the JAK2/STAT3 pathway, is currently ongoing in patients with recurrent GBM (NCT01904123).

Inhibiting MDSCs is under investigation, using several approaches that include induction of MDSC differentiation into DC, decreasing MDSC levels, and inhibiting MDSC function (148). One study in patients with recurrent GBM (NCT02669173) aims at targeting MDSC using low-dose capecitabine, a prodrug of 5-fluorouracil, which was shown to kill MDSC and restore antitumor T cell responses (149). Another way of MDSC depletion is the use of colony-stimulating factor 1 receptor (CSF1-R) inhibitors. CSF1-R is overexpressed by MDSC and TAMs in human glioma and its expression was shown to correlate with glioma grade (150, 151). It is involved in the recruitment of TAM and MDSC at the tumor site *via* interaction with CSF1 and is necessary for their survival. CSF1-R inhibition showed improved survival in a preclinical model of glioma, with reprograming of the TAM into pro-inflammatory cells (152). Use of the CSF1-R inhibitor PLX3397 as single agent in patients with recurrent GBM showed no improvement in survival (153), however, combination studies in preclinical models of melanoma demonstrated improvement of adoptive cell therapy, accompanied by reduction of tumor-infiltrating TAM and MDSC and augmentation of IFN-γ-secreting TILs (154), advocating for its use in combination therapies in humans. The same molecule is currently being tested in patients with newly diagnosed GBM (NCT01790503) and another trial using a CSF1-R antibody is ongoing in combination with the anti-PD1 antibody nivolumab in patients with advanced cancers including glioma (NCT02526017).

Finally, inhibition of Tregs is currently being investigated for tumors in the brain in one trial only, although initial studies using an anti-CD25 antibody to deplete Tregs in combination with an EGFRvIII peptide vaccine showed enhanced humoral response to the vaccine in patients receiving the antibody (155). In the ongoing trial, pp65 CMV mRNA-pulsed DCs are injected into a Td vaccine-pretreated site, with or without the anti-CD25 antibody basiliximab (NCT02366728).

At the moment, there are no trials targeting the tumor microenvironment in patients with PCNSL, although there is a rationale for their implementation (156).

## Immune Checkpoint Blockade Trials

There are now numerous clinical trials testing the efficacy of ICB antibodies for tumors arising in the brain including glioma and PCNSL. An important issue related to the use of ICB antibodies is the mutation load of the targeted malignancies. GBM do not possess a high rate of mutations (around 2.5 mutations per megabase^2^) (157), except for a particular hypermutated rare subtype (158), lowering the probability of neoepitope-specific immune responses that can be amplified by ICB antibodies. Thus, the efficacy of immune checkpoint inhibitors might be less impressive as compared to other malignancies, as immune checkpoint inhibitors have been shown to work best in highly mutated tumors, with a threshold of 100 mutations per exome (3.3 mutations per megabase) (40, 41). As a consequence, trials in GBM might need to use these molecules not as single agents, but rather in combination with other immunotherapeutic strategies. Regarding PCNSL, recent studies revealed a median mutation load around 6.6 mutations per megabase (159), suggesting that this malignancy could be targeted with ICB antibodies as single agents. A further issue for the use of ICB antibodies from tumors located in the brain is whether efficacy is linked to penetration of antibodies to the tumor site in the CNS. Since, even in the condition where a tumor is present, blood–brain barrier breakdown is only partial, access of antibodies to tumors in the CNS will definitely be less efficient than for tumors located in peripheral organs. Anti-CTLA4, and to some extend anti-PD1, might exert their effect while seeing T cells in the periphery. Indeed, it has been shown that anti-PD1 treatment affected the phenotype of PD1-expressing Tregs in the peripheral blood of nivolumab-treated GBM patients (NCT02017717) (160). Considering anti-PD-L1 antibodies (such as durvalumab currently being tested in patients with GBM, see below), they will certainly need to access the tumor to reach PD-L1-expressing tumor cells, but an effect of anti-PD-L1 on circulating myeloid cells cannot be excluded. Studies in glioma mouse models have demonstrated the efficacy of anti-CTLA4 and anti-PD1 antibodies (161, 162) and studies demonstrating efficacy of anti-PD-L1 antibodies confirmed interest of these targets but do not provide the formal proof than these antibodies are able to enter the brain (144, 163). Brain metastases from melanoma patients can be controlled by ICB antibodies, but with lower efficacy than metastases in extracerebral sites (164).

The number of clinical trials for GBM using anti-CTLA4, but mostly anti-PD1, has increased remarkably in the last 2 years. Indeed, the anti-CTLA4 antibody ipilimumab is being tested in combination with anti-PD1 in newly diagnosed (NCT02311920) and recurrent GBM patients (NCT02017717, in comparison with bevacizumab, Table 4). Rationale for investigating efficacy of multiple ICB antibodies originate from clinical studies in melanoma demonstrating higher efficacy of combination of anti-CTLA4 and anti-PD1 versus anti-CTLA-4 (165, 166) or either agent alone in PD-L1-negative patients (166), the limiting factor being however increased toxicity as treatments are combined. Preclinical studies also showed that only combination of ICB antibodies were able to induce regression of intracranial glioma (144, 161). Nevertheless, several trials using the anti-PD1 antibodies nivolumab (fully human IgG_4_), pembrolizumab (humanized IgG_4_), or pidilizumab (humanized IgG_1_) are ongoing in the adult and pediatric populations in pilot, phase I, II, and III trials. Some trials are investigating anti-PD1 antibodies as single agents in newly diagnosed (NCT02667587, NCT02617589, NCT02550249, NCT02530502) or recurrent (NCT02550249, NCT02313272, NCT02359565, NCT02337686, NCT02337491) GBM patients, including children (NCT02550249, NCT02359565, NCT01952769). One trial is comparing the use of pembrolizumab in comparison to three suppressors of the PI3K/Akt pathways given together (NCT02430363). Rare hypermutated GBM tumors occurring in patients suffering from biallelic mismatch repair deficiency, which have been shown to respond to nivolumab treatment (158), are being targeted as well (NCT02658279). Two trials are addressing the efficacy of other ICB, the anti-PD-L1 antibody durvalumab (human IgG_1_) in patients with newly diagnosed or recurrent GBM (NCT02336165) and an anti-LAG3 antibody compared to an anti-CD137 (urelumab, a fully human IgG4 antibody) combined or not with pembrolizumab (NCT02658981).

**Table 4:** **Currently ongoing immune checkpoint trials**.

Molecule	Additional intervention	Patient population	Diagnostic	Phase	Estimated enrollment	Country	NCT number
**Anti-CTLA4**							

Ipilimumab	±Nivolumab	Adult	Newly diagnosed glioblastoma (GBM)	I	42	USA	NCT02311920

Ipilimumab	Nivolumab	Adult	Recurrent GBM	III	440	Worldwide (checkmate 143)	NCT02017717

**Anti-PD1**							

Nivolumab	Gamma knife + valproate	Adult	Recurrent GBM	Pilot	17	USA	NCT02648633

Nivolumab	None and/or ipilimumab	Adult	Newly diagnosed GBM	I	42	USA	NCT02311920

Nivolumab	CSF1-R inhibitor	Adult	Solid tumors, GBM	I	270	USA	NCT02526017

Nivolumab	Galunisertib (TGFβ receptor I kinase inhibitor)	Adult	GBM, other solid tumors	I/II	100	USA and Spain	NCT02423343

Nivolumab		Adult	Newly diagnosed GBM (Me MGMT)	II randomized	320	Worldwide (checkmate 548)	NCT02667587

Nivolumab		Adult	Newly diagnosed GBM (UnMe MGMT)	III	550	Worldwide (checkmate 498)	NCT02617589

Nivolumab		Pediatric + adult	Newly diagnosed and recurrent GBM	II	29	Spain	NCT02550249

Nivolumab	CMV pp65-mRNA-pulsed dendritic cells	Adult	Recurrent HGG	II	66	USA	NCT02529072

Pembrolizumab		Adult	Recurrent HGG with hypermutant phenotype	Pilot	12	USA	NCT02658279

Pembrolizumab		Adult	Recurrent HGG	I	32	USA	NCT02313272

Pembrolizumab		Pediatric	Recurrent HGG/DPIG	I	70	USA	NCT02359565

Pembrolizumab		Adult	Newly diagnosed HGG	I/II	50	USA	NCT02530502

Pembrolizumab	MRI-guided laser ablation	Adult	Newly diagnosed HGG	I/II	52	USA	NCT02311582

Pembrolizumab		Adult	Recurrent GBM	II	20	USA	NCT02337686

Pembrolizumab		Adult	Recurrent GBM	II	81	USA	NCT02337491

Pembrolizumab		Adult	Recurrent PCNSL	II	21	Austria	NCT02779101

Pembrolizumab	Versus three PI3K/Akt pathways inhibitors	Adult	Recurrent GBM	IIb	58	Worldwide	NCT02430363

Pidilizumab		Pediatric	DPIG	I/II	50	Israel	NCT01952769

**Anti-PD-L1**							

Durvalumab	Bevacizumab	Adult	Newly diagnosed and recurrent GBM	II	108	USA and Australia	NCT02336165

**Anti-LAG3**							

Anti-LAG3	Pembrolizumab, urelumab	Adult	Recurrent GBM	I	68	USA	NCT02658981

*Durvalumab, human IgG1 anti-PD-L1; HGG, high-grade (III and IV) astrocytoma; ipilimumab, humanized IgG1 anti-CTLA4; Me MGMT, methylated MGMT promoter; nivolumab, fully human IgG4 anti-PD1; NSCLC, non-small cell lung cancer; PCNSL, primary CNS lymphoma; pembrolizumab, humanized IgG4 anti-PD1; pidilizumab, humanized IgG1 anti-PD1; urelumab, fully human IgG4 anti-CD137; UnMe MGMT, unmethylated MGMT promoter*.

Currently, none of these trials are selecting patients according to the PD-L1 status. It has been shown in non-CNS malignancies that response to PD1 targeting was associated with PD-L1 expression (167–169) and one study demonstrated objective responses in patients whose tumors expressed PD-L1 only (169). However, in contrast to this, some studies observed treatment responses in PD-L1-negative patients, questioning the use of PD-L1 expression as a marker for patient selection. In that matter, one issue is the various protocols (including different antibodies, tumor sample size, cut-offs…) used for the assessment of PD-L1 expression that prevents direct comparison of studies (170). Regarding GBM, the same issue applies, but, regardless of the methodology used, the rate of PD-L1-positive tumors seems to be relatively high as compared to non-CNS malignancies (171). Expression in PCNSL samples, although less intensively assessed thus far, seems to be lower (172, 173). A careful assessment of PD-L1 expression in ongoing clinical trials of anti-PD1 and PD-L1 will be invaluable in helping define the role of PD-L1 expression as a marker of treatment efficacy in CNS malignancies.

As mentioned before, the relatively low mutation load of GBM might require using ICB antibodies in combination with antitumor vaccines or other therapeutic interventions. In that regard, other studies are combining anti-PD1 antibodies with (i) approaches to enhance tumor immunogenicity, (ii) therapeutic vaccines, or (iii) molecules targeting the tumor microenvironment. Enhancement of tumor immunogenicity is achieved through the concomitant use of gamma knife surgery to provide additional tumor antigens to the immune system and valproic acid, a histone deacetylase inhibitor shown to induce global DNA demethylation (NCT02648633). Others are using peritumoral MRI-guided laser ablation in order to breach the BBB and increase access of tumor antigens to the immune system (NCT02311582). At the moment, only one trial combining ICB antibodies with another immunotherapy is ongoing, using autologous DCs pulsed with pp65 CMV mRNA (NCT02529072). As mentioned above, elicitation of antitumor immune responses that reach the tumor is associated with adaptive immune resistance as tumor infiltration by IFN-γ-secreting cells lead to upregulation to PD-L1 in the tumor environment (37), a phenomenon that could be counteracted in a glioma mouse model of tumor-loaded DC vaccination by the concomitant use of anti-PD1 antibodies (162). Therefore, combining DC and other vaccines with ICB antibodies certainly merits further exploration. As already mentioned above, two trials are using ICB in the context of strategies aiming at targeting the tumor microenvironment, namely using a CSF1-R inhibitor (NCT02526017) or a TGFβ receptor I kinase inhibitor (NCT02423343). Regarding PCNSL, one trial is currently addressing the effect of anti-PD1 antibodies in recurrent PCNSL (NCT02779101).

## Conclusion

Currently, ongoing trials for tumors located in the brain are principally designed on the same basis as for tumors located at other sites. Similarities between CNS and non-CNS tumors are the need for specificity, the need for T cell infiltration in the case of non-T cell inflamed organs, and the need to overcome local immunosuppression. The only feature that is unique to tumors located in the brain is the absence of metastases outside the CNS. This is an opportunity, as, if we can design immunotherapies that are efficient in getting functional antitumor T cell in the CNS, no other site needs to be targeted. Once we achieve this, the difference for tumors located in the brain will be determining the tolerated level for an inflammatory response to occur without damage to the brain. Integration of these parameters into future clinical trials will ultimately result in clinical benefit for the patient. In the interim, maximizing the biological information from existing trials may be highly informative. Finally, a notion that is also true for tumors located outside the brain, we should aim at investigating combination of vaccines, cell therapy, ICB antibodies, and molecules targeting the tumor environment, trying as well to exploit the beneficial effects of radio- and chemotherapy. In this regard, selecting patients according to markers such as mutational load, tumor PD-L1 expression, and extent of T cell infiltration might help define combinations most beneficial for each patient. Understanding whether a non-inflamed tumor is the result of tumor escape or immune ignorance will help choose between helping existing T cells to efficiently exert their antitumor effect, providing exogenous T cells, and inducing tumor cell death to provide antigens to the immune system. With this, the dream of immunotherapy might come true, with long-lasting tumor remissions without significant toxicity to be seen for improvement of patient survival.

## Author Contributions

All authors listed, have made substantial, direct and intellectual contribution to the work, and approved it for publication.

## Conflict of Interest Statement

P-YD reports receiving a research funding from immatics Biotechnologies GmbH. The other authors declare no conflict of interest.

## Footnotes

^1^http://www.celldex.com/pipeline/rindopepimut.php.

^2^http://icgc.org/.

## References

[1] Schreiber RD Old LJ Smyth MJ. Cancer immunoediting: integrating immunity’s roles in cancer suppression and promotion. Science (2011) 331:1565–70. 10.1126/science.1203486 21436444

[2] Walker PR Calzascia T de Tribolet N Dietrich PY T-cell immune responses in the brain and their relevance for cerebral malignancies. Brain Res Brain Res Rev (2003) 42:97–122. 10.1016/S0165-0173(03)00141-3 12738053

[3] Guo C Manjili MH Subjeck JR Sarkar D Fisher PB Wang XY. Therapeutic cancer vaccines: past, present, and future. Adv Cancer Res (2013) 119:421–75. 10.1016/B978-0-12-407190-2.00007-1 23870514 PMC3721379

[4] Gubin MM Artyomov MN Mardis ER Schreiber RD. Tumor neoantigens: building a framework for personalized cancer immunotherapy. J Clin Invest (2015) 125:3413–21. 10.1172/JCI80008 26258412 PMC4588307

[5] Hinrichs CS Restifo NP. Reassessing target antigens for adoptive T-cell therapy. Nat Biotechnol (2013) 31:999–1008. 10.1038/nbt.2725 24142051 PMC4280065

[6] Ohnmacht GA Wang E Mocellin S Abati A Filie A Fetsch P Short-term kinetics of tumor antigen expression in response to vaccination. J Immunol (2001) 167:1809–20. 10.4049/jimmunol.167.3.1809 11466407

[7] Sampson JH Heimberger AB Archer GE Aldape KD Friedman AH Friedman HS Immunologic escape after prolonged progression-free survival with epidermal growth factor receptor variant III peptide vaccination in patients with newly diagnosed glioblastoma. J Clin Oncol (2010) 28:4722–9. 10.1200/JCO.2010.28.6963 20921459 PMC3020702

[8] Ribas A Timmerman JM Butterfield LH Economou JS. Determinant spreading and tumor responses after peptide-based cancer immunotherapy. Trends Immunol (2003) 24:58–61. 10.1016/S1471-4906(02)00029-7 12547500

[9] Azad TD Razavi SM Jin B Lee K Li G Glioblastoma antigen discovery – foundations for immunotherapy. J Neurooncol (2015) 123:347–58. 10.1007/s11060-015-1836-8 26045361

[10] Cicchelero L de RH Sanders NN. Various ways to improve whole cancer cell vaccines. Expert Rev Vaccines (2014) 13:721–35. 10.1586/14760584.2014.911093 24758597

[11] Tagliamonte M Petrizzo A Tornesello ML Buonaguro FM Buonaguro L Antigen-specific vaccines for cancer treatment. Hum Vaccin Immunother (2014) 10:3332–46. 10.4161/21645515.2014.973317 25483639 PMC4514024

[12] Hofman FM Stathopoulos A Kruse CA Chen TC Schijns VE. Immunotherapy of malignant gliomas using autologous and allogeneic tissue cells. Anticancer Agents Med Chem (2010) 10:462–70. 10.2174/1871520611009060462 20879986 PMC3999913

[13] Pizzurro GA Barrio MM. Dendritic cell-based vaccine efficacy: aiming for hot spots. Front Immunol (2015) 6:91. 10.3389/fimmu.2015.00091 25784913 PMC4347494

[14] Rosenberg SA Yang JC Restifo NP. Cancer immunotherapy: moving beyond current vaccines. Nat Med (2004) 10:909–15. 10.1038/nm1100 15340416 PMC1435696

[15] Temizoz B Kuroda E Ishii KJ Vaccine adjuvants as potential cancer immunotherapeutics. Int Immunol (2016) 28:329–38. 10.1093/intimm/dxw015 27006304 PMC4922024

[16] Hanson MC Crespo MP Abraham W Moynihan KD Szeto GL Chen SH Nanoparticulate STING agonists are potent lymph node-targeted vaccine adjuvants. J Clin Invest (2015) 125:2532–46. 10.1172/JCI79915 25938786 PMC4497758

[17] Chandra D Quispe-Tintaya W Jahangir A Asafu-Adjei D Ramos I Sintim HO STING ligand c-di-GMP improves cancer vaccination against metastatic breast cancer. Cancer Immunol Res (2014) 2:901–10. 10.1158/2326-6066.CIR-13-0123 24913717 PMC4264585

[18] Ohkuri T Ghosh A Kosaka A Zhu J Ikeura M David M STING contributes to antiglioma immunity via triggering type I IFN signals in the tumor microenvironment. Cancer Immunol Res (2014) 2:1199–208. 10.1158/2326-6066.CIR-14-0099 25300859 PMC4258479

[19] Mitchell DA Batich KA Gunn MD Huang MN Sanchez-Perez L Nair SK Tetanus toxoid and CCL3 improve dendritic cell vaccines in mice and glioblastoma patients. Nature (2015) 519:366–9. 10.1038/nature14320 25762141 PMC4510871

[20] Harris DT Kranz DM Adoptive T cell therapies: a comparison of T cell receptors and chimeric antigen receptors. Trends Pharmacol Sci (2016) 37:220–30. 10.1016/j.tips.2015.11.004 26705086 PMC4764454

[21] Fridman WH Pages F Sautes-Fridman C Galon J. The immune contexture in human tumours: impact on clinical outcome. Nat Rev Cancer (2012) 12:298–306. 10.1038/nrc3245 22419253

[22] Rosenberg SA Restifo NP. Adoptive cell transfer as personalized immunotherapy for human cancer. Science (2015) 348:62–8. 10.1126/science.aaa4967 25838374 PMC6295668

[23] Debets R Donnadieu E Chouaib S Coukos G TCR-engineered T cells to treat tumors: seeing but not touching? Semin Immunol (2016) 28:10–21. 10.1016/j.smim.2016.03.002 26997556

[24] Gill S Maus MV Porter DL Chimeric antigen receptor T cell therapy: 25years in the making. Blood Rev (2015) 30:157–67. 10.1016/j.blre.2015.10.003 26574053

[25] Restifo NP Dudley ME Rosenberg SA. Adoptive immunotherapy for cancer: harnessing the T cell response. Nat Rev Immunol (2012) 12:269–81. 10.1038/nri3191 22437939 PMC6292222

[26] Casucci M Hawkins RE Dotti G Bondanza A. Overcoming the toxicity hurdles of genetically targeted T cells. Cancer Immunol Immunother (2015) 64:123–30. 10.1007/s00262-014-1641-9 25488419 PMC11028535

[27] Cameron BJ Gerry AB Dukes J Harper J Kannan V Bianchi FC Identification of a Titin-derived HLA-A1-presented peptide as a cross-reactive target for engineered MAGE A3-directed T cells. Sci Transl Med (2013) 5:197ra103 10.1126/scitranslmed.3006034 PMC6002776 23926201

[28] Barrett DM Teachey DT Grupp SA. Toxicity management for patients receiving novel T-cell engaging therapies. Curr Opin Pediatr (2014) 26:43–9. 10.1097/MOP.0000000000000043 24362408 PMC4198063

[29] Leisegang M Engels B Schreiber K Yew PY Kiyotani K Idel C Eradication of large solid tumors by gene therapy with a T-cell receptor targeting a single cancer-specific point mutation. Clin Cancer Res (2016) 22:2734–43. 10.1158/1078-0432.CCR-15-2361 26667491 PMC4891260

[30] Vignard V Lemercier B Lim A Pandolfino MC Guilloux Y Khammari A Adoptive transfer of tumor-reactive Melan-A-specific CTL clones in melanoma patients is followed by increased frequencies of additional Melan-A-specific T cells. J Immunol (2005) 175:4797–805. 10.4049/jimmunol.175.7.4797 16177129

[31] Chapuis AG Roberts IM Thompson JA Margolin KA Bhatia S Lee SM T-Cell therapy using interleukin-21-primed cytotoxic T-cell lymphocytes combined with cytotoxic T-cell lymphocyte antigen-4 blockade results in long-term cell persistence and durable tumor regression. J Clin Oncol (2016) 34:3787–95. 10.1200/JCO.2015.65.5142 PMC5477923 27269940

[32] Pitt JM Marabelle A Eggermont A Soria JC Kroemer G Zitvogel L. Targeting the tumor microenvironment: removing obstruction to anticancer immune responses and immunotherapy. Ann Oncol (2016) 27:1482–92. 10.1093/annonc/mdw168 27069014

[33] Takeuchi Y Nishikawa H. Roles of regulatory T cells in cancer immunity. Int Immunol (2016) 28:401–9. 10.1093/intimm/dxw025 27160722 PMC4986235

[34] Kim J Bae JS. Tumor-associated macrophages and neutrophils in tumor microenvironment. Mediators Inflamm (2016) 2016:6058147. 10.1155/2016/6058147 26966341 PMC4757693

[35] Malek E de LM Letterio JJ Kim BG Finke JH Driscoll JJ Myeloid-derived suppressor cells: the green light for myeloma immune escape. Blood Rev (2016) 30:341–8. 10.1016/j.blre.2016.04.002 27132116 PMC6411302

[36] Borch TH Donia M Andersen MH Svane IM. Reorienting the immune system in the treatment of cancer by using anti-PD-1 and anti-PD-L1 antibodies. Drug Discov Today (2015) 20:1127–34. 10.1016/j.drudis.2015.07.003 26189934

[37] Topalian SL Drake CG Pardoll DM. Immune checkpoint blockade: a common denominator approach to cancer therapy. Cancer Cell (2015) 27:450–61. 10.1016/j.ccell.2015.03.001 25858804 PMC4400238

[38] Dubinski D Wolfer J Hasselblatt M Schneider-Hohendorf T Bogdahn U Stummer W CD4+ T effector memory cell dysfunction is associated with the accumulation of granulocytic myeloid-derived suppressor cells in glioblastoma patients. Neuro Oncol (2016) 18:807–18. 10.1093/neuonc/nov280 26578623 PMC4864257

[39] Ito A Kondo S Tada K Kitano S. Clinical development of immune checkpoint inhibitors. Biomed Res Int (2015) 2015:605478. 10.1155/2015/605478 26161407 PMC4486755

[40] Snyder A Makarov V Merghoub T Yuan J Zaretsky JM Desrichard A Genetic basis for clinical response to CTLA-4 blockade in melanoma. N Engl J Med (2014) 371:2189–99. 10.1056/NEJMoa1406498 25409260 PMC4315319

[41] Le DT Uram JN Wang H Bartlett BR Kemberling H Eyring AD PD-1 blockade in tumors with mismatch-repair deficiency. N Engl J Med (2015) 372:2509–20. 10.1056/NEJMoa1500596 26028255 PMC4481136

[42] Robbins PF Lu YC El-Gamil M Li YF Gross C Gartner J Mining exomic sequencing data to identify mutated antigens recognized by adoptively transferred tumor-reactive T cells. Nat Med (2013) 19:747–52. 10.1038/nm.3161 23644516 PMC3757932

[43] Ansell SM Lesokhin AM Borrello I Halwani A Scott EC Gutierrez M PD-1 blockade with nivolumab in relapsed or refractory Hodgkin’s lymphoma. N Engl J Med (2015) 372:311–9. 10.1056/NEJMoa1411087 25482239 PMC4348009

[44] Romano E Michielin O Voelter V Laurent J Bichat H Stravodimou A MART-1 peptide vaccination plus IMP321 (LAG-3Ig fusion protein) in patients receiving autologous PBMCs after lymphodepletion: results of a Phase I trial. J Transl Med (2014) 12:97. 10.1186/1479-5876-12-97 24726012 PMC4021605

[45] Legat A Maby-El HH Baumgaertner P Cagnon L Abed Maillard S Geldhof C Vaccination with LAG-3Ig (IMP321) and peptides induces specific CD4 and CD8 T-cell responses in metastatic melanoma patients-report of a phase I/IIa clinical trial. Clin Cancer Res (2016) 22:1330–40. 10.1158/1078-0432.CCR-15-1212 26500235

[46] Butowski NA. Epidemiology and diagnosis of brain tumors. Continuum (Minneap Minn) (2015) 21:301–13. 10.1212/01.CON.0000464171.50638.fa 25837897

[47] Ostrom QT Gittleman H Fulop J Fulop J Liu M Blanda R CBTRUS statistical report: primary brain and central nervous system tumors diagnosed in the United States in 2008–2012. Neuro Oncol (2015) 17(Suppl 4):iv1–62. 10.1093/neuonc/nou327 26511214 PMC4623240

[48] Derouazi M Di Berardino-Besson W Belnoue E Hoepner S Walther R Benkhoucha M Novel cell-penetrating peptide-based vaccine induces robust CD4+ and CD8+ T cell-mediated antitumor immunity. Cancer Res (2015) 75:3020–31. 10.1158/0008-5472.CAN-14-3017 26116496

[49] Masson F Calzascia T Di Berardino-Besson W de Tribolet N Dietrich PY Walker PR. Brain microenvironment promotes the final functional maturation of tumor-specific effector CD8+ T cells. J Immunol (2007) 179:845–53. 10.4049/jimmunol.179.2.845 17617575

[50] Kim YH Jung TY Jung S Jang WY Moon KS Kim IY Tumour-infiltrating T-cell subpopulations in glioblastomas. Br J Neurosurg (2012) 26:21–7. 10.3109/02688697.2011.584986 21707245

[51] Kmiecik J Poli A Brons NH Waha A Eide GE Enger PØ Elevated CD3+ and CD8+ tumor-infiltrating immune cells correlate with prolonged survival in glioblastoma patients despite integrated immunosuppressive mechanisms in the tumor microenvironment and at the systemic level. J Neuroimmunol (2013) 264:71–83. 10.1016/j.jneuroim.2013.08.013 24045166

[52] Lohr J Ratliff T Huppertz A Ge Y Dictus C Ahmadi R Effector T-cell infiltration positively impacts survival of glioblastoma patients and is impaired by tumor-derived TGF-beta. Clin Cancer Res (2011) 17:4296–308. 10.1158/1078-0432.CCR-10-2557 21478334

[53] Herold-Mende C Mossemann J Jungk C Ahmadi R Unterberg A Capper D Peripheral T cell responses in glioblastoma patients are associated with an imprrved survival [abstract]. Neuro Oncol (2014) 16(Suppl2):ii13 10.1093/neuonc/nou209.1

[54] Pallasch CP Struss AK Munnia A König J Steudel WI Fischer U Autoantibodies against GLEA2 and PHF3 in glioblastoma: tumor-associated autoantibodies correlated with prolonged survival. Int J Cancer (2005) 117:456–9. 10.1002/ijc.20929 15906353

[55] Ueda R Low KL Zhu X Fujita M Sasaki K Whiteside TL Spontaneous immune responses against glioma-associated antigens in a long term survivor with malignant glioma. J Transl Med (2007) 5:68. 10.1186/1479-5876-5-68 18093336 PMC2244605

[56] Dutoit V Herold-Mende C Hilf N Schoor O Beckhove P Bucher J Exploiting the glioblastoma peptidome to discover novel tumour-associated antigens for immunotherapy. Brain (2012) 135:1042–54. 10.1093/brain/aws042 22418738

[57] Ponzoni M Berger F Chassagne-Clement C Tinguely M Jouvet A Ferreri AJ Reactive perivascular T-cell infiltrate predicts survival in primary central nervous system B-cell lymphomas. Br J Haematol (2007) 138:316–23. 10.1111/j.1365-2141.2007.06661.x 17555470

[58] He M Zuo C Wang J Liu J Jiao B Zheng J Prognostic significance of the aggregative perivascular growth pattern of tumor cells in primary central nervous system diffuse large B-cell lymphoma. Neuro Oncol (2013) 15:727–34. 10.1093/neuonc/not012 23482670 PMC3661096

[59] Cserr HF Knopf PM. Cervical lymphatics, the blood-brain barrier and the immunoreactivity of the brain: a new view. Immunol Today (1992) 13:507–12. 10.1016/0167-5699(92)90027-5 1463583

[60] Louveau A Smirnov I Keyes TJ Eccles JD Rouhani SJ Peske JD Structural and functional features of central nervous system lymphatic vessels. Nature (2015) 523:337–41. 10.1038/nature14432 26030524 PMC4506234

[61] Ransohoff RM Engelhardt B. The anatomical and cellular basis of immune surveillance in the central nervous system. Nat Rev Immunol (2012) 12:623–35. 10.1038/nri3265 22903150

[62] Calzascia T Masson F Di Berardino-Besson W Contassot E Wilmotte R Aurrand-Lions M Homing phenotypes of tumor-specific CD8 T cells are predetermined at the tumor site by crosspresenting APCs. Immunity (2005) 22:175–84. 10.1016/j.immuni.2004.12.008 15723806

[63] Okada H. Brain tumor immunotherapy with type-1 polarizing strategies. Ann N Y Acad Sci (2009) 1174:18–23. 10.1111/j.1749-6632.2009.04932.x 19769732

[64] Carbone FR. Tissue-resident memory T cells and fixed immune surveillance in nonlymphoid organs. J Immunol (2015) 195:17–22. 10.4049/jimmunol.1500515 26092813

[65] Facoetti A Nano R Zelini P Morbini P Benericetti E Ceroni M Human leukocyte antigen and antigen processing machinery component defects in astrocytic tumors. Clin Cancer Res (2005) 11:8304–11. 10.1158/1078-0432.CCR-04-2588 16322289

[66] Han J Alvarez-Breckenridge CA Wang QE Yu J TGF-beta signaling and its targeting for glioma treatment. Am J Cancer Res (2015) 5:945–55. 26045979 PMC4449428

[67] Reardon DA Turner S Peters KB Desjardins A Gururangan S Sampson JH A review of VEGF/VEGFR-targeted therapeutics for recurrent glioblastoma. J Natl Compr Canc Netw (2011) 9:414–27. 21464146 10.6004/jnccn.2011.0038 PMC3399727

[68] Sawamura Y Diserens AC de TN. In vitro prostaglandin E2 production by glioblastoma cells and its effect on interleukin-2 activation of oncolytic lymphocytes. J Neurooncol (1990) 9:125–30. 10.1007/BF02427832 2175768

[69] Rolle CE Sengupta S Lesniak MS. Mechanisms of immune evasion by gliomas. Adv Exp Med Biol (2012) 746:53–76. 10.1007/978-1-4614-3146-6_5 22639159

[70] Wainwright DA Balyasnikova IV Chang AL Ahmed AU Moon KS Auffinger B IDO expression in brain tumors increases the recruitment of regulatory T cells and negatively impacts survival. Clin Cancer Res (2012) 18:6110–21. 10.1158/1078-0432.CCR-12-2130 22932670 PMC3500434

[71] Kohanbash G Okada H. Myeloid-derived suppressor cells (MDSCs) in gliomas and glioma-development. Immunol Invest (2012) 41:658–79. 10.3109/08820139.2012.689591 23017140

[72] Ooi YC Tran P Ung N Thill K Trang A Fong BM The role of regulatory T-cells in glioma immunology. Clin Neurol Neurosurg (2014) 119:125–32. 10.1016/j.clineuro.2013.12.004 24582432

[73] Gabrusiewicz K Rodriguez B Wei J Hashimoto Y Healy LM Maiti SN Glioblastoma-infiltrated innate immune cells resemble M0 macrophage phenotype. JCI Insight (2016) 1:e85841 10.1172/jci.insight.85841 26973881 PMC4784261

[74] Saas P Walker PR Hahne M Quiquerez AL Schnuriger V Perrin G Fas ligand expression by astrocytoma in vivo: maintaining immune privilege in the brain? J Clin Invest (1997) 99:1173–8. 10.1172/JCI119273 9077524 PMC507930

[75] Bloch O Crane CA Kaur R Safaee M Rutkowski MJ Parsa AT. Gliomas promote immunosuppression through induction of B7-H1 expression in tumor-associated macrophages. Clin Cancer Res (2013) 19:3165–75. 10.1158/1078-0432.CCR-12-3314 23613317 PMC3742575

[76] Wintterle S Schreiner B Mitsdoerffer M Schneider D Chen L Meyermann R Expression of the B7-related molecule B7-H1 by glioma cells: a potential mechanism of immune paralysis. Cancer Res (2003) 63:7462–7. 14612546

[77] Berghoff AS Kiesel B Widhalm G Rajky O Ricken G Wöhrer A Programmed death ligand 1 expression and tumor-infiltrating lymphocytes in glioblastoma. Neuro Oncol (2015) 17:1064–75. 10.1093/neuonc/nou307 25355681 PMC4490866

[78] Evans SM Judy KD Dunphy I Jenkins WT Hwang WT Nelson PT Hypoxia is important in the biology and aggression of human glial brain tumors. Clin Cancer Res (2004) 10:8177–84. 10.1158/1078-0432.CCR-04-1081 15623592

[79] Perng P Lim M. Immunosuppressive mechanisms of malignant gliomas: parallels at non-CNS sites. Front Oncol (2015) 5:153. 10.3389/fonc.2015.00153 26217588 PMC4492080

[80] Galea I Bechmann I Perry VH. What is immune privilege (not)? Trends Immunol (2007) 28:12–8. 10.1016/j.it.2006.11.004 17129764

[81] Thomas DL Kranz DM Roy EJ. Experimental manipulations of afferent immune responses influence efferent immune responses to brain tumors. Cancer Immunol Immunother (2008) 57:1323–33. 10.1007/s00262-008-0467-8 18278494 PMC11030392

[82] Macarthur KM Kao GD Chandrasekaran S Alonso-Basanta M Chapman C Lustig RA Detection of brain tumor cells in the peripheral blood by a telomerase promoter-based assay. Cancer Res (2014) 74:2152–9. 10.1158/0008-5472.CAN-13-0813 24525740 PMC4144786

[83] Muller C Holtschmidt J Auer M Heitzer E Lamszus K Schulte A Hematogenous dissemination of glioblastoma multiforme. Sci Transl Med (2014) 6:247ra101 10.1126/scitranslmed.3009095 25080476

[84] Ohlfest JR Andersen BM Litterman AJ Xia J Pennell CA Swier LE Vaccine injection site matters: qualitative and quantitative defects in CD8 T cells primed as a function of proximity to the tumor in a murine glioma model. J Immunol (2013) 190:613–20. 10.4049/jimmunol.1201557 23248259 PMC3538956

[85] Hickey WF. Leukocyte traffic in the central nervous system: the participants and their roles. Semin Immunol (1999) 11:125–37. 10.1006/smim.1999.0168 10329499

[86] Hong JJ Rosenberg SA Dudley ME Yang JC White DE Butman JA Successful treatment of melanoma brain metastases with adoptive cell therapy. Clin Cancer Res (2010) 16:4892–8. 10.1158/1078-0432.CCR-10-1507 20719934 PMC6291850

[87] Kluger HM Zito CR Barr ML Baine MK Chiang VL Sznol M Characterization of PD-L1 expression and associated T-cell infiltrates in metastatic melanoma samples from variable anatomic sites. Clin Cancer Res (2015) 21:3052–60. 10.1158/1078-0432.CCR-14-3073 25788491 PMC4490112

[88] Nishimura F Dusak JE Eguchi J Zhu X Gambotto A Storkus WJ Adoptive transfer of type 1 CTL mediates effective anti-central nervous system tumor response: critical roles of IFN-inducible protein-10. Cancer Res (2006) 66:4478–87. 10.1158/0008-5472.CAN-05-3825 16618775

[89] Jack CS Arbour N Manusow J Montgrain V Blain M McCrea E TLR signaling tailors innate immune responses in human microglia and astrocytes. J Immunol (2005) 175:4320–30. 10.4049/jimmunol.175.7.4320 16177072

[90] Okada H Kalinski P Ueda R Hoji A Kohanbash G Donegan TE Induction of CD8+ T-cell responses against novel glioma-associated antigen peptides and clinical activity by vaccinations with {alpha}-type 1 polarized dendritic cells and polyinosinic-polycytidylic acid stabilized by lysine and carboxymethylcellulose in patients with recurrent malignant glioma. J Clin Oncol (2011) 29:330–6. 10.1200/JCO.2010.30.7744 21149657 PMC3056467

[91] Okada H Butterfield LH Hamilton RL Hoji A Sakaki M Ahn BJ Induction of robust type-I CD8+ T-cell responses in WHO grade 2 low-grade glioma patients receiving peptide-based vaccines in combination with poly-ICLC. Clin Cancer Res (2015) 21:286–94. 10.1158/1078-0432.CCR-14-1790 25424847 PMC4297523

[92] Craddock JA Lu A Bear A Pule M Brenner MK Rooney CM Enhanced tumor trafficking of GD2 chimeric antigen receptor T cells by expression of the chemokine receptor CCR2b. J Immunother (2010) 33:780–8. 10.1097/CJI.0b013e3181ee6675 20842059 PMC2998197

[93] Hoepner S Walker PR Getting by with a little help from the right CD4 T cells. Oncoimmunology (2013) 2:e25772 10.4161/onci.25772 24244904 PMC3825730

[94] Bottner M Krieglstein K Unsicker K. The transforming growth factor-betas: structure, signaling, and roles in nervous system development and functions. J Neurochem (2000) 75:2227–40. 10.1046/j.1471-4159.2000.0752227.x 11080174

[95] Voron T Marcheteau E Pernot S Colussi O Tartour E Taieb J Control of the immune response by pro-angiogenic factors. Front Oncol (2014) 4:70. 10.3389/fonc.2014.00070 24765614 PMC3980099

[96] Bechmann I Mor G Nilsen J Eliza M Nitsch R Naftolin F FasL (CD95L, Apo1L) is expressed in the normal rat and human brain: evidence for the existence of an immunological brain barrier. Glia (1999) 27:62–74. 10.1002/(SICI)1098-1136(199907)27:1<62::AID-GLIA7>3.0.CO;2-S 10401633

[97] Woo SR Corrales L Gajewski TF. The STING pathway and the T cell-inflamed tumor microenvironment. Trends Immunol (2015) 36:250–6. 10.1016/j.it.2015.02.003 25758021 PMC4393801

[98] Taube JM Anders RA Young GD Xu H Sharma R McMiller TL Colocalization of inflammatory response with B7-h1 expression in human melanocytic lesions supports an adaptive resistance mechanism of immune escape. Sci Transl Med (2012) 4:127ra37 10.1126/scitranslmed.3003689 PMC3568523 22461641

[99] Spranger S Spaapen RM Zha Y Williams J Meng Y Ha TT Up-regulation of PD-L1, IDO, and T(regs) in the melanoma tumor microenvironment is driven by CD8(+) T cells. Sci Transl Med (2013) 5:200ra116. 10.1126/scitranslmed.3006504 23986400 PMC4136707

[100] Ulloa-Montoya F Louahed J Dizier B Gruselle O Spiessens B Lehmann FF Predictive gene signature in MAGE-A3 antigen-specific cancer immunotherapy. J Clin Oncol (2013) 31:2388–95. 10.1200/JCO.2012.44.3762 23715562

[101] Topalian SL Hodi FS Brahmer JR Gettinger SN Smith DC McDermott DF Safety, activity, and immune correlates of anti-PD-1 antibody in cancer. N Engl J Med (2012) 366:2443–54. 10.1056/NEJMoa1200690 22658127 PMC3544539

[102] Tumeh PC Harview CL Yearley JH Shintaku IP Taylor EJ Robert L PD-1 blockade induces responses by inhibiting adaptive immune resistance. Nature (2014) 515:568–71. 10.1038/nature13954 25428505 PMC4246418

[103] Gajewski TF Schreiber H Fu YX. Innate and adaptive immune cells in the tumor microenvironment. Nat Immunol (2013) 14:1014–22. 10.1038/ni.2703 24048123 PMC4118725

[104] Chang C Lin CH Cheng AL Medeiros LJ Chang KC. Primary central nervous system diffuse large B-cell lymphoma has poorer immune cell infiltration and prognosis than its peripheral counterpart. Histopathology (2015) 67:625–35. 10.1111/his.12706 25829022

[105] Burnette BC Liang H Lee Y Chlewicki L Khodarev NN Weichselbaum RR The efficacy of radiotherapy relies upon induction of type I interferon-dependent innate and adaptive immunity. Cancer Res (2011) 71:2488–96. 10.1158/0008-5472.CAN-10-2820 21300764 PMC3070872

[106] Deng L Liang H Xu M Yang X Burnette B Arina A STING-dependent cytosolic DNA sensing promotes radiation-induced type I interferon-dependent antitumor immunity in immunogenic tumors. Immunity (2014) 41:843–52. 10.1016/j.immuni.2014.10.019 25517616 PMC5155593

[107] Tang H Wang Y Chlewicki LK Zhang Y Guo J Liang W Facilitating T cell infiltration in tumor microenvironment overcomes resistance to PD-L1 blockade. Cancer Cell (2016) 29:285–96. 10.1016/j.ccell.2016.02.004 26977880 PMC4794755

[108] Okada H Kohanbash G Zhu X Kastenhuber ER Hoji A Ueda R Immunotherapeutic approaches for glioma. Crit Rev Immunol (2009) 29:1–42. 10.1615/CritRevImmunol.v29.i1.10 19348609 PMC2713019

[109] Overwijk WW Wang E Marincola FM Rammensee HG Restifo NP. Mining the mutanome: developing highly personalized Immunotherapies based on mutational analysis of tumors. J Immunother Cancer (2013) 1:11. 10.1186/2051-1426-1-11 24829748 PMC4019909

[110] Padfield E Ellis HP Kurian KM. Current therapeutic advances targeting EGFR and EGFRvIII in glioblastoma. Front Oncol (2015) 5:5. 10.3389/fonc.2015.00005 25688333 PMC4310282

[111] Ichimura K Narita Y Hawkins CE. Diffusely infiltrating astrocytomas: pathology, molecular mechanisms and markers. Acta Neuropathol (2015) 129:789–808. 10.1007/s00401-015-1439-7 25975377

[112] Pollack IF Jakacki RI Butterfield LH Hamilton RL Panigrahy A Normolle OP Immune responses and outcome after vaccination with glioma-associated antigen peptides and poly-ICLC in a pilot study for pediatric recurrent low-grade gliomasdagger. Neuro Oncol (2016) 18:1157–68. 10.1007/s11060-016-2245-3 26984745 PMC4933485

[113] Rampling R Peoples S Mulholland PJ James A Al-Salihi O Twelves CJ A cancer research UK first time in human phase I trial of IMA950 (novel multi peptide therapeutic vaccine) in patients with newly diagnosed glioblastoma. Clin Cancer Res (2016) 22:4776–85. 10.1158/1078-0432.CCR-16-0506 27225692 PMC5026298

[114] Iwami K Shimato S Ohno M Okada H Nakahara N Sato Y Peptide-pulsed dendritic cell vaccination targeting interleukin-13 receptor alpha2 chain in recurrent malignant glioma patients with HLA-A*24/A*02 allele. Cytotherapy (2012) 14:733–42. 10.3109/14653249.2012.666633 22424217

[115] Hashimoto N Tsuboi A Kagawa N Chiba Y Izumoto S Kinoshita M Wilms tumor 1 peptide vaccination combined with temozolomide against newly diagnosed glioblastoma: safety and impact on immunological response. Cancer Immunol Immunother (2015) 64:707–16. 10.1007/s00262-015-1674-8 25772149 PMC11028974

[116] Sakai K Shimodaira S Maejima S Udagawa N Sano K Higuchi Y Dendritic cell-based immunotherapy targeting Wilms’ tumor 1 in patients with recurrent malignant glioma. J Neurosurg (2015) 123:989–97. 10.3171/2015.1.JNS141554 26252465

[117] Sampson JH Aldape KD Archer GE Coan A Desjardins A Friedman AH Greater chemotherapy-induced lymphopenia enhances tumor-specific immune responses that eliminate EGFRvIII-expressing tumor cells in patients with glioblastoma. Neuro Oncol (2011) 13:324–33. 10.1093/neuonc/noq157 21149254 PMC3064599

[118] Schuster J Lai RK Recht LD Reardon DA Paleologos NA Groves MD A phase II, multicenter trial of rindopepimut (CDX-110) in newly diagnosed glioblastoma: the ACT III study. Neuro Oncol (2015) 17:854–61. 10.1093/neuonc/nou348 25586468 PMC4483122

[119] Belnoue E Berardino-Besson WD Gaertner H Carboni S Dunand-Sauthier I Cerini F Enhancing anti-tumor immune responses by optimized combinations of cell-penetrating peptide based-vaccines and adjuvants. Mol Ther (2016) 24:1675–85. 10.1038/mt.2016.134 27377043 PMC5113103

[120] Hailemichael Y Dai Z Jaffarzad N Ye Y Medina MA Huang XF Persistent antigen at vaccination sites induces tumor-specific CD8(+) T cell sequestration, dysfunction and deletion. Nat Med (2013) 19:465–72. 10.1038/nm.3105 23455713 PMC3618499

[121] Grauer OM Molling JW Bennink E Toonen LW Sutmuller RP Nierkens S TLR ligands in the local treatment of established intracerebral murine gliomas. J Immunol (2008) 181:6720–9. 10.4049/jimmunol.181.10.6720 18981089

[122] Tchoghandjian A Baeza-Kallee N Beclin C Metellus P Colin C Ducray F Cortical and subventricular zone glioblastoma-derived stem-like cells display different molecular profiles and differential in vitro and in vivo properties. Ann Surg Oncol (2012) 19(Suppl 3):S608–19. 10.1245/s10434-011-2093-5 21989663

[123] Quattrocchi KB Miller CH Cush S Bernard SA Dull ST Smith M Pilot study of local autologous tumor infiltrating lymphocytes for the treatment of recurrent malignant gliomas. J Neurooncol (1999) 45:141–57. 10.1023/A:1006293606710 10778730

[124] Lawler SE. Cytomegalovirus and glioblastoma; controversies and opportunities. J Neurooncol (2015) 123:465–71. 10.1007/s11060-015-1734-0 25682092

[125] Dix AR Brooks WH Roszman TL Morford LA. Immune defects observed in patients with primary malignant brain tumors. J Neuroimmunol (1999) 100:216–32. 10.1016/S0165-5728(99)00203-9 10695732

[126] Jackson HJ Rafiq S Brentjens RJ. Driving CAR T-cells forward. Nat Rev Clin Oncol (2016) 13:370–83. 10.1038/nrclinonc.2016.36 27000958 PMC5529102

[127] Gill S June CH. Going viral: chimeric antigen receptor T-cell therapy for hematological malignancies. Immunol Rev (2015) 263:68–89. 10.1111/imr.12243 25510272

[128] Kahlon KS Brown C Cooper LJ Raubitschek A Forman SJ Jensen MC. Specific recognition and killing of glioblastoma multiforme by interleukin 13-zetakine redirected cytolytic T cells. Cancer Res (2004) 64:9160–6. 10.1158/0008-5472.CAN-04-0454 15604287

[129] Morgan RA Yang JC Kitano M Dudley ME Laurencot CM Rosenberg SA. Case report of a serious adverse event following the administration of T cells transduced with a chimeric antigen receptor recognizing ERBB2. Mol Ther (2010) 18:843–51. 10.1038/mt.2010.24 20179677 PMC2862534

[130] Caruso HG Hurton LV Najjar A Rushworth D Ang S Olivares S Tuning sensitivity of CAR to EGFR density limits recognition of normal tissue while maintaining potent antitumor activity. Cancer Res (2015) 75:3505–18. 10.1158/0008-5472.CAN-15-0139 26330164 PMC4624228

[131] Tugues S Burkhard SH Ohs I Vrohlings M Nussbaum K Vom Berg J New insights into IL-12-mediated tumor suppression. Cell Death Differ (2015) 22:237–46. 10.1038/cdd.2014.134 25190142 PMC4291488

[132] Berger C Jensen MC Lansdorp PM Gough M Elliott C Riddell SR. Adoptive transfer of effector CD8+ T cells derived from central memory cells establishes persistent T cell memory in primates. J Clin Invest (2008) 118:294–305. 10.1172/JCI32103 18060041 PMC2104476

[133] Roy LO Poirier MB Fortin D. Transforming growth factor-beta and its implication in the malignancy of gliomas. Target Oncol (2015) 10:1–14. 10.1007/s11523-014-0308-y 24590691

[134] Bogdahn U Hau P Stockhammer G Venkataramana NK Mahapatra AK Suri A Targeted therapy for high-grade glioma with the TGF-beta2 inhibitor trabedersen: results of a randomized and controlled phase IIb study. Neuro Oncol (2011) 13:132–42. 10.1093/neuonc/noq142 20980335 PMC3018908

[135] Brandes AA Carpentier AF Kesari S Sepulveda-Sanchez JM Wheeler HR Chinot O A phase II randomized study of galunisertib monotherapy or galunisertib plus lomustine compared with lomustine monotherapy in patients with recurrent glioblastoma. Neuro Oncol (2016) 18:1146–56. 10.1093/neuonc/now009 26902851 PMC4933481

[136] den Hollander MW Bensch F Glaudemans AW Oude Munnink TH Enting RH den Dunnen WF TGF-beta antibody uptake in recurrent high-grade glioma imaged with 89Zr-fresolimumab PET. J Nucl Med (2015) 56:1310–4. 10.2967/jnumed.115.154401 26135113

[137] Deharvengt S Marmarelis M Korc M. Concomitant targeting of EGF receptor, TGF-beta and SRC points to a novel therapeutic approach in pancreatic cancer. PLoS One (2012) 7:e39684. 10.1371/journal.pone.0039684 22761868 PMC3384603

[138] Friedman HS Prados MD Wen PY Mikkelsen T Schiff D Abrey LE Bevacizumab alone and in combination with irinotecan in recurrent glioblastoma. J Clin Oncol (2009) 27:4733–40. 10.1200/JCO.2008.19.8721 19720927

[139] Kreisl TN Kim L Moore K Duic P Royce C Stroud I Phase II trial of single-agent bevacizumab followed by bevacizumab plus irinotecan at tumor progression in recurrent glioblastoma. J Clin Oncol (2009) 27:740–5. 10.1200/JCO.2008.16.3055 19114704 PMC2645088

[140] Chinot OL Wick W Mason W Henriksson R Saran F Nishikawa R Bevacizumab plus radiotherapy-temozolomide for newly diagnosed glioblastoma. N Engl J Med (2014) 370:709–22. 10.1056/NEJMoa1308345 24552318

[141] Gilbert MR Dignam JJ Armstrong TS Wefel JS Blumenthal DT Vogelbaum MA A randomized trial of bevacizumab for newly diagnosed glioblastoma. N Engl J Med (2014) 370:699–708. 10.1056/NEJMoa1308573 24552317 PMC4201043

[142] de Groot JF Lamborn KR Chang SM Gilbert MR Cloughesy TF Aldape K Phase II study of aflibercept in recurrent malignant glioma: a North American Brain Tumor Consortium study. J Clin Oncol (2011) 29:2689–95. 10.1200/JCO.2010.34.1636 21606416 PMC3139373

[143] Roy EJ Takikawa O Kranz DM Brown AR Thomas DL. Neuronal localization of indoleamine 2,3-dioxygenase in mice. Neurosci Lett (2005) 387:95–9. 10.1016/j.neulet.2005.07.010 16076525

[144] Wainwright DA Chang AL Dey M Balyasnikova IV Kim CK Tobias A Durable therapeutic efficacy utilizing combinatorial blockade against IDO, CTLA-4, and PD-L1 in mice with brain tumors. Clin Cancer Res (2014) 20:5290–301. 10.1158/1078-0432.CCR-14-0514 24691018 PMC4182350

[145] Abou-Ghazal M Yang DS Qiao W Reina-Ortiz C Wei J Kong LY The incidence, correlation with tumor-infiltrating inflammation, and prognosis of phosphorylated STAT3 expression in human gliomas. Clin Cancer Res (2008) 14:8228–35. 10.1158/1078-0432.CCR-08-1329 19088040 PMC2605668

[146] See AP Han JE Phallen J Binder Z Gallia G Pan F The role of STAT3 activation in modulating the immune microenvironment of GBM. J Neurooncol (2012) 110:359–68. 10.1007/s11060-012-0981-6 23096132 PMC3700337

[147] Komohara Y Horlad H Ohnishi K Ohta K Makino K Hondo H M2 macrophage/microglial cells induce activation of Stat3 in primary central nervous system lymphoma. J Clin Exp Hematop (2011) 51:93–9. 10.3960/jslrt.51.93 22104307

[148] Najjar YG Finke JH. Clinical perspectives on targeting of myeloid derived suppressor cells in the treatment of cancer. Front Oncol (2013) 3:49. 10.3389/fonc.2013.00049 23508517 PMC3597982

[149] Vincent J Mignot G Chalmin F Ladoire S Bruchard M Chevriaux A 5-Fluorouracil selectively kills tumor-associated myeloid-derived suppressor cells resulting in enhanced T cell-dependent antitumor immunity. Cancer Res (2010) 70:3052–61. 10.1158/0008-5472.CAN-09-3690 20388795

[150] Komohara Y Horlad H Ohnishi K Fujiwara Y Bai B Nakagawa T Importance of direct macrophage-tumor cell interaction on progression of human glioma. Cancer Sci (2012) 103:2165–72. 10.1111/cas.12015 22957741 PMC7659278

[151] Bender AM Collier LS Rodriguez FJ Tieu C Larson JD Halder C Sleeping beauty-mediated somatic mutagenesis implicates CSF1 in the formation of high-grade astrocytomas. Cancer Res (2010) 70:3557–65. 10.1158/0008-5472.CAN-09-4674 20388773 PMC2862088

[152] Pyonteck SM Akkari L Schuhmacher AJ Bowman RL Sevenich L Quail DF CSF-1R inhibition alters macrophage polarization and blocks glioma progression. Nat Med (2013) 19:1264–72. 10.1038/nm.3337 24056773 PMC3840724

[153] Butowski N Colman H De Groot JF Omuro AM Nayak L Wen PY Orally administered colony stimulating factor 1 receptor inhibitor PLX3397 in recurrent glioblastoma: an Ivy Foundation Early Phase Clinical Trials Consortium phase II study. Neuro Oncol (2016) 18:557–64. 10.1093/neuonc/nov245 26449250 PMC4799682

[154] Mok S Koya RC Tsui C Xu J Robert L Wu L Inhibition of CSF-1 receptor improves the antitumor efficacy of adoptive cell transfer immunotherapy. Cancer Res (2014) 74:153–61. 10.1158/0008-5472.CAN-13-1816 24247719 PMC3947337

[155] Sampson JH Schmittling RJ Archer GE Congdon KL Nair SK Reap EA A pilot study of IL-2Ralpha blockade during lymphopenia depletes regulatory T-cells and correlates with enhanced immunity in patients with glioblastoma. PLoS One (2012) 7:e31046 10.1371/journal.pone.0031046 22383993 PMC3288003

[156] Ponzoni M Issa S Batchelor TT Rubenstein JL. Beyond high-dose methotrexate and brain radiotherapy: novel targets and agents for primary CNS lymphoma. Ann Oncol (2014) 25:316–22. 10.1093/annonc/mdt385 24265352 PMC3905778

[157] Alexandrov LB Nik-Zainal S Wedge DC Aparicio SA Behjati S Biankin AV Signatures of mutational processes in human cancer. Nature (2013) 500:415–21. 10.1038/nature12477 23945592 PMC3776390

[158] Bouffet E Larouche V Campbell BB Merico D de Borja R Aronson M Immune checkpoint inhibition for hypermutant glioblastoma multiforme resulting from germline biallelic mismatch repair deficiency. J Clin Oncol (2016) 34:2206–11. 10.1200/JCO.2016.66.6552 27001570

[159] Vater I Montesinos-Rongen M Schlesner M Haake A Purschke F Sprute R The mutational pattern of primary lymphoma of the central nervous system determined by whole-exome sequencing. Leukemia (2015) 29:677–85. 10.1038/leu.2014.264 25189415

[160] Lowther DE Goods BA Lucca LE Lerner BA Raddassi K van Dijk D PD-1 marks dysfunctional regulatory T cells in malignant gliomas. JCI Insight (2016) 1:e85935 10.1172/jci.insight.85935 27182555 PMC4864991

[161] Kim JE Patel MA Mangraviti A Kim ES Theodros D Velarde E Combination therapy with anti-PD-1, anti-TIM-3, and focal radiation results in regression of murine gliomas. Clin Cancer Res (2016). 10.1158/1078-0432.CCR-15-1535 27358487 PMC5735836

[162] Antonios JP Soto H Everson RG Orpilla J Moughon D Shin N PD-1 blockade enhances the vaccination-induced immune response in glioma. JCI Insight (2016) 1:e87059. 10.1172/jci.insight.87059 27453950 PMC4951098

[163] Reardon DA Gokhale PC Klein SR Ligon KL Rodig SJ Ramkissoon SH Glioblastoma eradication following immune checkpoint blockade in an orthotopic, immunocompetent model. Cancer Immunol Res (2016) 4:124–35. 10.1158/2326-6066.CIR-15-0151 26546453

[164] Ajithkumar T Parkinson C Fife K Corrie P Jefferies S. Evolving treatment options for melanoma brain metastases. Lancet Oncol (2015) 16:e486–97. 10.1016/S1470-2045(15)00141-2 26433822

[165] Postow MA Chesney J Pavlick AC Robert C Grossmann K McDermott D Nivolumab and ipilimumab versus ipilimumab in untreated melanoma. N Engl J Med (2015) 372:2006–17. 10.1056/NEJMoa1414428 25891304 PMC5744258

[166] Larkin J Chiarion-Sileni V Gonzalez R Grob JJ Cowey CL Lao CD Combined nivolumab and ipilimumab or monotherapy in untreated melanoma. N Engl J Med (2015) 373:23–34. 10.1056/NEJMoa1504030 26027431 PMC5698905

[167] Garon EB Rizvi NA Hui R Leighl N Balmanoukian AS Eder JP Pembrolizumab for the treatment of non-small-cell lung cancer. N Engl J Med (2015) 372:2018–28. 10.1056/NEJMoa1501824 25891174

[168] Carbognin L Pilotto S Milella M Vaccaro V Brunelli M Calio A Differential activity of nivolumab, pembrolizumab and MPDL3280A according to the tumor expression of programmed death-ligand-1 (PD-L1): sensitivity analysis of trials in melanoma, lung and genitourinary cancers. PLoS One (2015) 10:e0130142 10.1371/journal.pone.0130142 26086854 PMC4472786

[169] Topalian SL Sznol M McDermott DF Kluger HM Carvajal RD Sharfman WH Survival, durable tumor remission, and long-term safety in patients with advanced melanoma receiving nivolumab. J Clin Oncol (2014) 32:1020–30. 10.1200/JCO.2013.53.0105 24590637 PMC4811023

[170] Patel SP Kurzrock R PD-L1 expression as a predictive biomarker in cancer immunotherapy. Mol Cancer Ther (2015) 14:847–56. 10.1158/1535-7163.MCT-14-0983 25695955

[171] Preusser M Berghoff AS Wick W Weller M. Clinical neuropathology mini-review 6-2015: PD-L1: emerging biomarker in glioblastoma? Clin Neuropathol (2015) 34:313–21. 10.5414/NP300922 26501438 PMC4766797

[172] Chapuy B Roemer MG Stewart C Tan Y Abo RP Zhang L Targetable genetic features of primary testicular and primary central nervous system lymphomas. Blood (2016) 127:869–81. 10.1182/blood-2015-10-673236 26702065 PMC4760091

[173] Berghoff AS Ricken G Widhalm G Rajky O Hainfellner JA Birner P PD1 (CD279) and PD-L1 (CD274, B7H1) expression in primary central nervous system lymphomas (PCNSL). Clin Neuropathol (2014) 33:42–9. 10.5414/NP300698 24359606

